# Perirhinal cortex abnormalities impair hippocampal plasticity and learning in *Scn2a*, *Fmr1*, and *Cdkl5* autism mouse models

**DOI:** 10.1126/sciadv.adt0780

**Published:** 2025-03-07

**Authors:** Rachel E. Keith, Yiming Shen, Jordan A. Janzen-Meza, Joseph Abramovitz, Priscila C. Antonello, Aysha Hameed, Baskar Mohana Krishnan, Michelle W. Antoine

**Affiliations:** ^1^ Section on Neural Circuits, National Institute on Alcohol Abuse and Alcoholism, National Institutes of Health, Bethesda, MD, USA.; ^2^ Washington University School of Medicine, St. Louis, MO, USA.; ^3^ George Mason University, Fairfax, VA, USA.

## Abstract

Learning and memory deficits, including spatial navigation difficulties, are common in autism spectrum disorder (ASD). Several ASD mouse models (*Scn2a^+/−^*, *Fmr1^−/−^*, *Cdkl5^−/−^*) exhibit impaired spatial learning, with these deficits often attributed to hippocampal dysfunction. However, we identify the perirhinal cortex (PRC) as a critical driver of these deficits. Cortical-wide *Scn2a* reduction in excitatory neurons replicated the spatial learning and long-term potentiation (LTP) impairments—a cellular correlate of learning—seen in *Scn2a^+/−^* mice, while hippocampal-wide reduction did not. PRC-specific viral-mediated *Scn2a* reduction in excitatory neurons decreased release probability, which consequently disrupted synaptic transmission and LTP in the hippocampus, as well as spatial learning. As PRC activity was reduced, chemogenetic activation of the PRC reversed these deficits in *Scn2a^+/−^* mice and rescued spatial learning and LTP impairments in *Fmr1* and *Cdkl5* knockout mice. Thus, in several genetic models of ASD, PRC abnormalities may disrupt hippocampal function to impair learning and memory.

## Teaser

In autism, dysfunction of the perirhinal cortex can impair learning, memory, and the ability to navigate unfamiliar environments.

## INTRODUCTION

Autism spectrum disorder (ASD) is a neurodevelopmental condition marked by atypical social behaviors, sensory processing, and cognitive function. Cognitive atypicalities in ASD individuals often involve difficulties with relational binding—connecting items to their contexts. This skill is crucial for spatial navigation, interpreting social cues, and engaging in reciprocal interactions (*1*). Children with ASD frequently display reduced visual-spatial abilities (*1*) and struggle with spatial navigation (*2*–*5*). The neurobiological mechanisms underlying these spatial deficits may intersect with those involved in social impairments.

ASD exhibits substantial genetic heterogeneity, with over 100 de novo gene mutations linked to increased risk (*6*). One notable gene, *SCN2A*, which encodes *Na_v_1.2*, the type II α subunit of the voltage-gated sodium channel, is among the strongest single gene candidates for autism susceptibility (*6*–*8*). In both mice and humans, heterozygous loss-of-function (LOF) mutations in *SCN2A* reduce gene expression and sodium current densities by ~50% (*9*–*11*). In humans, these mutations are linked to ASD with intellectual disability (ID) with a penetrance of up to 50%, and spatial memory issues, including disorientation and difficulty remembering object locations (*8*). In mice and humans, homozygosity for LOF *Scn2a* mutations is incompatible with survival (*11*) and *Scn2a* heterozygous knockout mice (*Scn2a^+/−^*) model ASD features, including impaired spatial learning (*12*, *13*).

LOF X-linked mutations in fragile X messenger ribonucleoprotein 1 (*FMR1*) and cyclin-dependent kinase–like 5 (*CDKL5*) are also associated with ASD and ID. *FMR1* mutations are the largest single-gene cause of ASD with ID, affecting 5% of ASD patients (*14*), while *CDKL5* mutations are rare (*15*, *16*). *Fmr1* knockout (*Fmr1 KO*) and *Cdkl5* knockout (*Cdkl5 KO*) mice recapitulate several patient features, including spatial processing deficits (*17*–*20*). Despite the association between LOF mutations in *SCN2A*, *FMR1*, and *CDKL5* and ASD with ID, shared mechanisms underlying cognitive impairments remain unknown. This study aims to identify overlapping brain regions and neural circuits affected by these genes and how these neural changes affect cognitive functions like spatial navigation.

In mice, spatial navigation can be evaluated using behavioral paradigms similar to human testing (*21*, *22*). Here, we use the dryland-based Barnes maze test (*12*, *21*, *22*). Studies on ASD mouse models often link spatial learning deficits to reduced long-term potentiation (LTP) at the hippocampal Schaffer collateral–CA1 (SC-CA1) pathway (*13*, *23*–*26*). However, some mice show spatial learning impairments without disruptions in hippocampal LTP or long-term depression (LTD) (*27*–*29*). This suggests that in some genetic cases of ASD, spatial deficits may arise from disruptions in brain regions outside the hippocampus, or that hippocampal plasticity might be dispensable for spatial learning.

We used whole-brain light-sheet imaging for cFos, conditional genetics, electrophysiological measurements of neural activity, and chemogenetic activity manipulations in *Scn2a^+/−^* mice to identify brain regions and changes causing spatial navigation deficits. We generalized our findings to the *Fmr1* and *Cdkl5* KO mice. Consistent with previous studies, we confirmed deficits in spatial learning and SC-CA1 LTP (*12*, *13*). However, spatial learning deficits were due to reduced *Scn2a* in layer 2/3 (L2/3) excitatory pyramidal neurons (PNs) of the perirhinal cortex (PRC), reducing PRC activity and altering input-output (I-O) dynamics in the SC-CA1, impairing theta-burst LTP. Chemogenetic activation of PRC excitatory PNs rescued these impairments and spatial learning behavior. Similar deficits were seen in *Fmr1* and *Cdkl5* KO mice, and chemogenetic activation of PRC excitatory PNs was also effective. Overall, our study underscores the pivotal role of the PRC in the emergence of ASD and associated learning impairments.

## RESULTS

### *Scn2a^+/−^* mice display impaired spatial learning and cortical hypoactivity

**Fig. 1.**
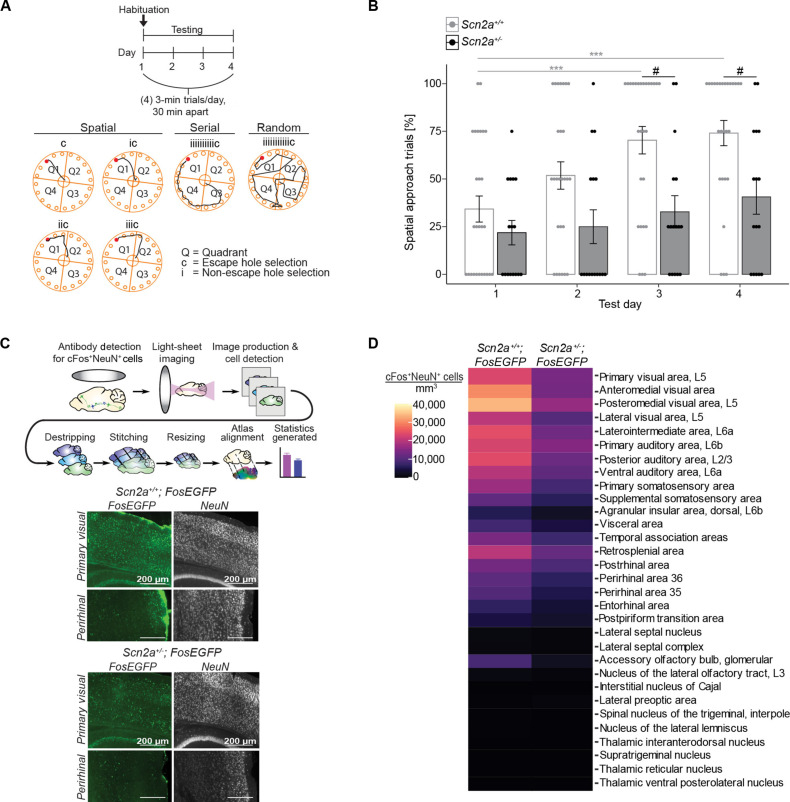
*Scn2a^+/−^* mice display impaired spatial learning and cortical hypoactivity. (**A**) Barnes maze (BM) method for evaluating spatial learning and memory. Highlighted are the prevalent navigation strategies—spatial, serial, or random—used by mice to locate the sole escape hole (depicted in red) among 19 other shallow holes. Maze quadrants are labeled Q1 to Q4. Correct hole selections are marked “c” and incorrect ones are marked “i.” (**B**) Scatter bar plot showing the percentage of spatial search trials in *Scn2a^+/−^* (*n* = 16) and *Scn2a^+/+^* (*n* = 27) mice across test days in the BM. Points represent individual mice; error bars indicate mean ± SEM. For within-subjects comparisons, a Friedman test (nonparametric one-way ANOVA) with Dunn’s post hoc test was used, whereas for between-subjects comparisons, a two-way RM ANOVA with a Tukey post hoc test was used. Friedman test: *P* < 0.0001 (*Scn2a^+/+^*) and *P* = 0.2434 (*Scn2a^+/−^*), ***(gray) *P* = 0.0003 and *P* = 0.0001; RM ANOVA: effect of genotype: *P* = 0.0161; effect of test day: *P* < 0.0001; ^#^(black) *P* = 0.0355 and *P* = 0.0490. (**C**) Whole-brain light-sheet imaging processing pipeline for cFos protein expression, a marker of cellular activity in neurons. Below are representative images of cFos-positive cells in the visual and perirhinal cortices of *Scn2a^+/+^;Fos*EGFP** and *Scn2a^+/−^;FosEGFP* mice. (**D**) Heatmap illustrating brain regions with significantly different cFos-positive neuron densities (*P* < 0.05) between *Scn2a^+/+^;FosEGFP* (*n* = 3) and *Scn2a^+/−^;FosEGFP* (*n* = 3) mouse brains. Unpaired two-tailed *t* tests at α = 0.05.

The Barnes maze is a widely used dryland test for evaluating spatial learning in rodents (*22*). In the 4-day training phase, consisting of four 3-min trials per day with a 30-min intertrial interval, mice learn to navigate among 19 shallow holes to locate and exit through an escape hole (Fig. 1A). Mice use one of three navigation strategies to find the escape hole: random, serial, or spatial search (*22*). The limit for incorrect selections that can still be categorized as spatial is three; thus, three or fewer erroneous selections are designated as spatial. For a detailed explanation of this criterion, please refer to Materials and Methods. Random searches occur during the initial one to two trials on the first day of testing, where mice cross the maze haphazardly to find the escape hole (Fig. 1A). Serial searches involve mice inspecting each hole within two or more quadrants to locate the escape hole, while spatial searches mainly focus on the quadrant containing the escape hole or move directly to it (Fig. 1A). Applying these criteria, we show that wild-type (*Scn2a^+/+^*) mice learn to locate the escape hole by test days 3 and 4, while mutant (*Scn2a^+/−^*) mice show significantly fewer spatial trials and no performance improvement (Fig. 1B). Male and female wild-type mice exhibit normal spatial learning, while *Scn2a^+/−^* mice of both sexes show similar deficits (fig. S1, A and B).

Consistent with spatial learning deficits in mutant mice, *Scn2a^+/+^* mice reduced the number of errors (selecting non-escape holes) made over three to four testing days, whereas *Scn2a^+/−^* mice did not (fig. S1, C and D). At the start of the task, mice often dart from the center of the maze into a hole, possibly perceiving it as a safe starting point. After pausing for a variable period, they begin selecting holes to search for the escape route. Over the four training days, escape latency can improve as mice either reduce the time taken to initiate the search or become more efficient at locating the escape hole. Notably, scoring of errors and spatial approach trials is unaffected by the latency to initiate the task. *Scn2a^+/+^* and *Scn2a^+/−^* mice show similar reductions in escape latency over time (fig. S1E). Although *Scn2a^+/−^* mice reduce their latency, their inability to reduce errors or significantly increase spatial approach trials suggests impaired spatial navigation. Therefore, using spatial pathway analysis may help improve the detection of spatial navigation deficits in certain cases. These findings replicate previous research showing impaired spatial learning in *Scn2a^+/−^* mice (*12*, *13*) and validate pathway analysis in the Barnes maze to detect these deficits.

*Scn2a* is expressed in glutamatergic neurons in the neocortex, hippocampus, and cerebellum, as well as in GABAergic projection neurons of the striatum (*30*). Its encoded protein, the α subunit of *Na_v_1.2*, functions in action potential initiation and propagation in young neurons and later facilitates somatodendritic excitability and action potential backpropagation (*31*–*33*). Given the potential for LOF *Scn2a* mutations to reduce neural circuit activity, we used whole-brain light-sheet imaging to examine expression of cFos, a marker of neuronal activity (*34*), to identify brain regions with significant activity changes contributing to the spatial learning impairment in *Scn2a^+/−^* mice (Fig. 1C). We generated mice with the cFos reporter (*Fos-EGFP*) on wild-type and *Scn2a^+/−^* backgrounds and then quantified neurons positive for cFos (cFos^+^NeuN^+^) to pinpoint affected brain areas. We identified 31 brain regions with reduced cFos^+^NeuN^+^ densities in *Scn2a^+/−^;FosEGFP* mice compared to the controls (*Scn2a^+/+^;FosEGFP* mice) (Fig. 1D). NeuN^+^ densities were comparable across these 31 brain regions (fig. S2A). The impacted areas were primarily cortical, with no observable alterations within the hippocampus (fig. S2B), despite this region being the most extensively studied area in spatial navigation literature (*35*). This aligns with previous in vitro physiological recordings showing normal excitability in *Scn2a^+/−^* hippocampal CA1 PNs (*13*). These results suggest that cortical hypoactivity may underlie the spatial learning impairment in *Scn2a^+/−^* mice.

### Decreased *Scn2a* levels in cortical PNs impaired spatial learning and reduced cFos protein expression

**Fig. 2.**
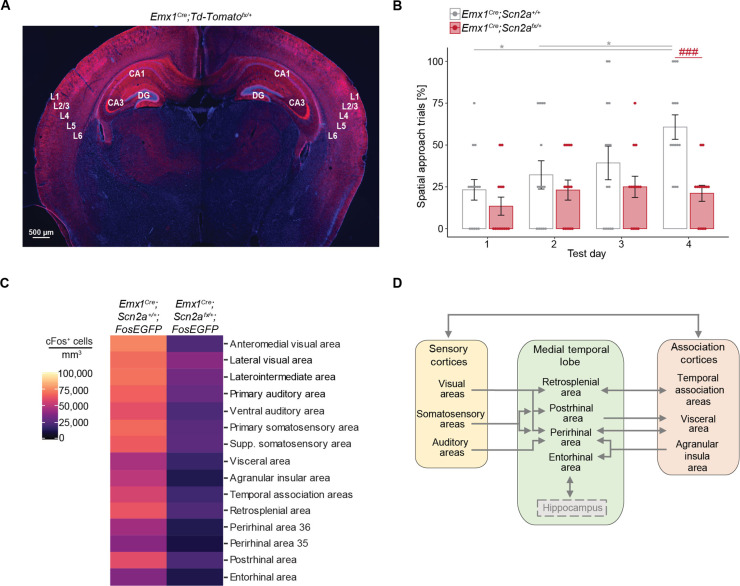
Decreased *Scn2a* levels in cortical PNs lead to impaired spatial learning and reduced cFos protein expression. (**A**) Brain image depicting Emx1^Cre^ recombination (red staining) in the cerebral cortex and hippocampus of *Emx1^Cre^;Td-Tomato^fx/+^* reporter mice. DAPI acts as a blue nuclear counterstain. (**B**) Scatter bar plot showing the percentage of spatial search trials in *Emx1^Cre^;Scn2a^fx/+^* (*n* = 13) and *Emx1^Cre^;Scn2a^+/+^* (*n* = 14) mice across test days in the BM. Points represent individual mice; error bars indicate mean ± SEM. Friedman test with Dunn’s post hoc test and two-way RM ANOVA with Tukey’s post hoc test. Friedman test: *P* = 0.0050 (*Emx1^Cre^;Scn2a^+/+^*) and *P* = 0.4914 (*Emx1^Cre^;Scn2a^fx/+^*), *(gray) *P* = 0.0259 in both cases; RM ANOVA: effect of genotype: *P* = 0.0083; effect of test day: *P* = 0.0082; ^###^*P* = 0.0006. (**C**) Heatmap showing 15 brain regions with significantly reduced cFos-positive cell densities (*P* < 0.05) between *Emx1^Cre^;Scn2a^fx/+^* (*n* = 3) and *Emx1^Cre^;Scn2a^+/+^* (*n* = 3) mice. (**D**) Putative network for spatial processing. Unpaired two-tailed *t* tests at α = 0.05.

As most (21 of 31) hypoactive areas were cortical, we generated mice with the *Emx1^Cre^* allele (*36*) and a tissue-specific **Scn2a*-*conditional *floxed* (*Scn2a^fx/+^*) allele (*13*) to facilitate Cre-mediated reduction of *Scn2a* expression in the cortex and hippocampus (Fig. 2A). This approach is designed to replicate the heterozygous *Scn2a^+/−^* mice, where there is a reduction of *Scn2a* in excitatory PNs in the cortical regions identified in the cFos dataset in Fig. 1D. *Emx1^Cre^;Scn2a^+/+^* mice displayed intact spatial learning, whereas *Emx1^Cre/+^*;*Scn2a^fx/+^* mice failed to learn the task by day 4 (Fig. 2A). Moreover, whole-brain light-sheet imaging to examine cFos in *Emx1^Cre^;Scn2a^fx/+^;FosEGFP* and *Emx1^Cre^;Scn2a^+/+^;FosEGFP* mice revealed hypoactivity in 15 of the 21 cortical areas (Fig. 2C) identified in the *Scn2a^+/−^* mice in Fig. 1D, with no changes in the hippocampus. This suggests that in the 15 brain areas, reduced *Scn2a* in cortical PNs decreases neural activity, contributing to the spatial learning deficit.

The medial temporal lobe (MTL) includes several key cortical structures involved in memory formation and spatial cognition, including the hippocampus. In line with the recapitulation of spatial learning defects, in both *Scn2a^+/−^* and *Emx1^Cre^;Scn2a^fx/+^* mice, four of five MTL cortical areas (retrosplenial, postrhinal, perirhinal, and entorhinal) showed diminished cFos expression (Figs. 1 and 2C). On the basis of established circuit connections to the MTL (*37*–*40*), we organized these 15 brain areas into a putative network for spatial processing (Fig. 2D). In this network, hypoactivity within one or more areas could potentially drive the spatial learning impairment.

In addition to excitatory PNs in the cortex, *Scn2a* is expressed in somatostatin-positive (SST) and vasoactive intestinal peptide–positive (VIP), but not parvalbumin-positive, inhibitory neurons of the neocortex (*30*). To rule out the potential role of loss of *Scn2a* in interneurons driving the spatial learning deficit, we generated *Vip^Cre^;Scn2a^fx/+^* and *SST^Cre^;Scn2a^fx/+^* mice (fig. S3). Both *Vip^Cre^;Scn2a^fx/+^* and *SST^Cre^;Scn2a^fx/+^* mice displayed normal spatial learning, while *Emx1^Cre^;Scn2a^fx/+^* mice did not (fig. S3), indicating that the loss of *Scn2a* in excitatory PNs specifically drives the spatial learning deficits in *Scn2a^+/−^* mice.

### Reducing *Scn2a* in the hippocampus does not impair spatial learning or SC-CA1 LTP

**Fig. 3.**
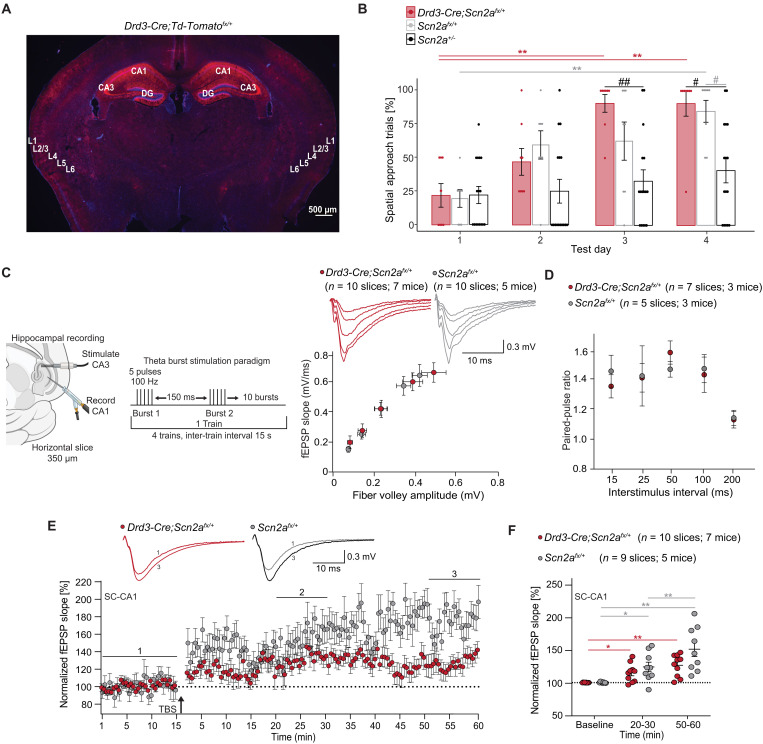
Reducing hippocampal *Scn2a* does not impair spatial learning or hippocampal physiology. (**A**) Brain image depicting *Drd3-Cre* recombination (red staining) in the cerebral cortex and hippocampus of *Drd3-Cre;Td-Tomato^fx/+^* reporter mice, with DAPI (blue stain) as a nuclear counterstain. (**B**) Scatter bar plot illustrates the percentage of spatial search trials in *Drd3-Cre;Scn2a^fx/+^* (*n* = 8), *Scn2a^fx/+^* mice (*n* = 8), and *Scn2a^+/−^* mice (*n* = 16) across test days in the BM. Points represent individual mice; error bars indicate mean ± SEM. Friedman test with Dunn’s post hoc test and two-way RM ANOVA with Tukey’s post hoc test. (**C**) Recording paradigm at the Schaffer collateral to CA1 synapse of the hippocampus (SC-CA1). The I-O relationship between FV and fEPSP slope in *Drd3-Cre;Scn2a^fx/+^* and *Scn2a^fx/+^* slices. Two-way RM ANOVA with Holm-Sidak post hoc test. (**D**) Paired-pulse ratio at the SC-CA1 synapse from *Drd3-Cre;Scn2a^fx/+^* and *Scn2a^fx/+^* slices. Two-way RM ANOVA with Fisher’s LSD post hoc test. (**E**) Inset shows representative fEPSPs before (1, baseline) and after TBS (3, 50 to 60 min) at SC-CA1. Plot of average normalized fEPSP slope over time in brain slices from *Drd3-Cre;Scn2a^fx/+^* and *Scn2a^fx/+^* mice. (**F**) Mean normalized fEPSP slope plot at baseline, 20 to 30 min, and 50 to 60 min after TBS. Each point represents an individual brain slice; bars denote mean ± SEM. Effect of time: *P* < 0.0001; interaction between time and genotype: *P* = 0.0436; **(gray) *P* = 0.0045 and *P* = 0.0058; *(gray) *P* = 0.0348, **(red) *P* = 0.0021; *(red) *P* = 0.0252.

As the cFos dataset showed no alterations in the hippocampus, we hypothesized that the spatial learning deficit in the *Emx1^Cre^;Scn2a^fx/+^* mice was primarily due to diminished *Scn2a* levels in the cortex. To determine whether *Scn2a* loss in the hippocampus alone impairs spatial learning, we generated tissue-specific *Scn2a-*conditional *floxed* (*Scn2a^fx/+^*) mice with the *Drd3-Cre* allele, which is expressed throughout the hippocampus and some cortical L2/3 intertelencephalic corticostriatal neurons (*41*) (Fig. 3A). *Drd3-Cre;Scn2a^fx/+^* mice exhibited a similar increase in spatial trials over time compared to wild-type mice (*Scn2a^fx/+^*) [Friedman’s test: *P* = 0.0013 (*Scn2a^fx/+^*); *P* < 0.0001 (*Drd3Cre;Scn2a^fx/+^*); Dunn’s post hoc test: **(gray) *P* = 0.0020, **(red) *P* = 0.0014 and *P* = 0.0020] (Fig. 3B). Furthermore, there were no significant differences in the number of spatial trials performed each day between *Drd3Cre;Scn2a^fx/+^* and *Scn2a^fx/+^* mice, suggesting similar learning curves [repeated-measures (RM) analysis of variance (ANOVA): effect of genotype: *P* = 0.0321; effect of test day: *P* < 0.0001; interaction: *P* = 0.0025; Tukey post hoc test: ^##^(black) *P* = 0.0017, ^#^(black) *P* = 0.0164, ^#^(gray) *P* = 0.0293]. These findings indicate that hippocampal reduction of *Scn2a* is not sufficient to recapitulate the spatial learning deficit observed in *Scn2a^+/−^* mice.

LTP strengthens synapses in response to neuronal stimulation (*25*, *26*). The SC-CA1 pathway, which consists of projections from CA3 to CA1, plays a key role in spatial memory encoding (*42*, *43*). We performed extracellular field recordings to evaluate theta-burst stimulation (TBS)-induced LTP (four trains every 15 s, each train comprising five pulses at 100 Hz with 150-ms interburst interval) in the SC-CA1 pathway of *Drd3-Cre;Scn2a^fx/+^* mice (Fig. 3C). In vitro TBS-LTP is closely associated with learning, as it replicates the in vivo physiological state of SC-CA1 during spatial navigation (*44*, *45*).

Basal synaptic transmission and paired-pulse facilitation at the SC-CA1 pathway were also unchanged in these mice (Fig. 3, C and D). As spatial learning was preserved in the *Drd3-Cre;Scn2a^fx/+^* mice, we expected that these mice would exhibit unaltered LTP. Consistent with preserved spatial learning, *Drd3-Cre;Scn2a^fx/+^* mice showed largely intact TBS-LTP compared to controls (Fig. 3, E and F, and Table 1). Previous work has shown intact basal synaptic transmission and LTD, but reduced TBS-LTP, at SC-CA1 in *Scn2a^+/−^* mice (*13*). This suggests that reduction in hippocampal LTP observed in *Scn2a^+/−^* mice (*13*) is not primarily due to the reduction of *Scn2a* in the hippocampus but instead originates from other brain regions such as the cortex.

**Table 1.** Raw data for LTP at the SC-CA1 synapse at the time points outlined in Fig. 3F.

*Drd3-Cre;Scn2a* ^*fx/+*^										
Baseline	99.76028	99.88678	99.63493	99.56797	99.91852	100.5046	99.83205	100.365	99.62518	100.4961
30 min	102.2262	96.27084	107.3424	121.6551	100.2758	116.3124	135.9612	119.8194	143.6849	119.1903
60 min	100.3566	123.9715	110.8043	138.0482	139.1375	130.7729	145.7356	146.2879	150.6444	105.1531
*Scn2a* ^*fx/+*^										
Baseline	99.05971	100.054	99.72788	99.48805	98.93315	101.1561	102.1271	100.6778	99.94614	
30 min	124.9373	157.68	115.3053	108.8697	162.0971	112.2934	141.5305	88.13306	125.2581	
60 min	133.5434	187.4891	169.5952	126.0705	215.7509	118.9087	193.1971	111.0963	148.9991	

### A global reduction of *Scn2a* in the cortex is associated with impaired PRC LTP and spatial learning

The hippocampus and adjacent cortical MTL structures are recognized as essential for the formation of long-term memory (*46*, *47*). However, it may be that the hippocampus supports certain memory functions distinct from those of the adjacent cortex. To enhance our understanding of the cortex's role in spatial memory, we generated *Scn2a^fx/+^* mice with the retinol binding protein 4 (*Rbp4*)–Cre allele, expressed in L5 cortical neurons as well as in ~30% of granule cells of the dentate gyrus (*41*, *48*) (fig. S4A). We also generated *Scn2a^fx/+^* mice with the neurotensin receptor type 1 (*Ntsr1*)–Cre allele, expressed in L6 cortical neurons (*41*) (fig. S4B). Both *Rbp4-Cre;Scn2a^fx/+^* and *Ntsr1-Cre;Scn2a^fx/+^* mice exhibited normal spatial learning (fig. S4, C and D). Further analysis of the spatial pattern of Cre recombination in the conditional *Scn2a^fx/+^* mutants containing *Emx1^Cre^*, *Drd3-Cre*, *Rbp4-Cre*, and *Ntsr1-Cre* alleles, along with an assessment of their spatial learning, identified the PRC as the MTL brain region most likely accountable for the spatial learning deficits (Table 2). Specifically, recombination in L2/3 of area 36 of PRC (formerly known as ectorhinal cortex) was only seen in the *Emx1^Cre^* line, the only line to recapitulate the spatial learning deficit (Table 2).

**Table 2.** Incidence of Cre recombination in the *Td-Tomato^fx/+^* reporter in mice with *Drd3-Cre*, *Rbp4-Cre*, *Ntsr1-Cre*, and Emx1*^Cre^* alleles. Recombination in brain regions was categorized as strong (++), partial (+), or none (−) based on *Td-Tomato* expression. *n* = 3 animals per genotype. Only the *Emx1^Cre^;Td-Tomato^fx/+^* mice showed recombination in the ectorhinal/PRC area 36.

Region	Genotype			
	*Emx1* ^*Cre*^	*Drd3-Cre*	*Rbp4-Cre*	*Ntsr1-Cre*
Anteromedial visual cortex				
Layer 2/3	++	+	−	−
Layer 5	++	+	++	−
Layer 6	++	+	−	++
Lateral visual				
Layer 2/3	++	+	−	−
Layer 5	++	+	++	−
Layer 6	++	+	−	++
Laterointermediate				
Layer 2/3	++	+	−	−
Layer 5	++	+	++	−
Layer 6	++	+	−	++
Primary auditory				
Layer 2/3	++	++	−	−
Layer 5	++	+	++	−
Layer 6	++	+	−	++
Ventral auditory				
Layer 2/3	++	++	−	−
Layer 5	++	+	++	−
Layer 6	++	+	−	++
Primary somatosensory				
Layer 2/3	++	++	−	−
Layer 5	++	+	++	−
Layer 6	++	+	−	++
Supplemental somatosensory				
Layer 2/3	++	++	−	−
Layer 5	++	++	++	−
Layer 6	++	++	−	++
Visceral				
Layer 2/3	++	+	−	−
Layer 5	++	+	−	−
Layer 6	++	+	−	+
Agranular insula				
Layer 2/3	++	+	−	−
Layer 5	++	+	−	−
Layer 6	++	+	−	+
Temporal association				
Layer 2/3	++	+	−	−
Layer 5	++	+	++	−
Layer 6	++	+	−	++
Retrosplenial				
Layer 2/3	++	+	−	−
Layer 5	++	+	++	−
Layer 6	++	+	−	−
Perirhinal (area 36)				
Layer 2/3	++	−	−	−
Layer 5	++	+	+	−
Layer 6	++	−	−	+
Perirhinal (area 35)				
Layer 2/3	++	+	−	−
Layer 5	++	+	+	−
Layer 6	++	+	−	−
Postrhinal				
Layer 2/3	+	++	−	−
Layer 5	+	++	+	−
Layer 6	+	+	−	−
Entorhinal				
Layer 2/3	+	+	−	−
Layer 5	+	+	+	−
Layer 6	+	+	−	−

Previous studies have indicated functional roles for the PRC that could influence hippocampal function and spatial learning performance, such as synchronization with hippocampal theta rhythm and maintenance of place cell stability (*49*, *50*). Furthermore, the PRC has projections to CA1 and CA3 (*51*, *52*), suggesting that this region may directly regulate hippocampal activity to influence spatial learning, potentially by modulating SC-CA1 plasticity. To assess this possibility, we evaluated basal synaptic transmission and TBS-LTP in L2/3 of the PRC using brain slices from control *Emx1^Cre^;Scn2a^+/+^* mice, which show intact spatial learning, and *Emx1^Cre^;Scn2a^+/−^* mice, which exhibit impaired spatial learning (fig. S5). Slices from both *Emx1^Cre^;Scn2a^+/+^* and *Emx1^Cre^;Scn2a^+/−^* mice showed normal basal synaptic transmission, with no overall genotype differences observed in the I-O curves for field excitatory postsynaptic potential (fEPSP) slope (fig. S5A). * Emx1^Cre^;Scn2a^+/−^* mice also displayed minimal to no TBS-LTP compared to controls (fig. S5, B and C). These results suggest that *Scn2a* in the PRC may be a crucial factor for LTP involved in spatial learning.

### PRC knockdown of *Scn2a* impairs PRC plasticity, spatial learning, and SC-CA1 LTP

**Fig. 4.**
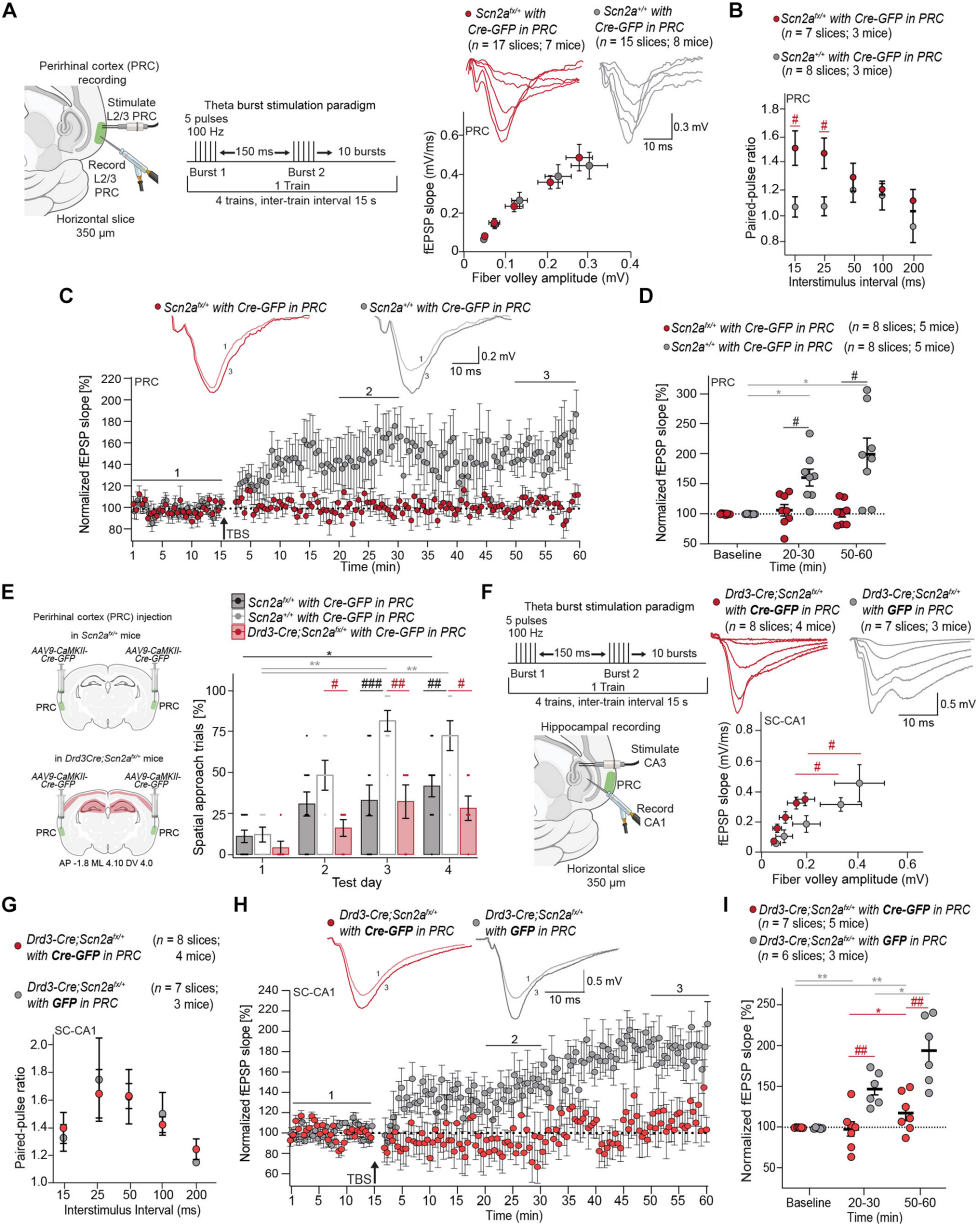
*Scn2a* reduction in the PRC impairs spatial learning, PRC L2/3 physiology, and hippocampal physiology. (**A**) Left: Recording paradigm in PRC. Right: I-O curve for *Scn2a^fx/+^* and wild-type littermates (*Scn2a^+/+^*) with *Cre-GFP* in PRC mice. Inset includes representative fEPSPs. Two-way RM ANOVA with Holm-Sidak post hoc test. (**B**) Paired-pulse ratio in the PRC from *Scn2a^fx/+^* and *Scn2a^+/+^* with *Cre-GFP* in PRC. Two-way RM ANOVA with Fisher’s LSD post hoc test. (**C**) Inset includes representative fEPSPs before (1, baseline) and after TBS (3, 50 to 60 min) in PRC. Plot of normalized fEPSP slope over time in slices from *Scn2a^fx/+^* and *Scn2a^+/+^* mice with *Cre-GFP* in PRC. (**D**) Mean normalized fEPSP slope plot at baseline, 20 to 30 min, and 50 to 60 min after TBS. (**E**) Left: Approach for *AAV9-CaMKII-Cre-GFP* viral-mediated reduction of *Scn2a* in the PRC in *Scn2a^fx/+^* mice. Right: Scatter bar plot showing the percentage of spatial search trials in *Scn2a^fx/+^* (*n* = 11), *Scn2a^+/+^* (*n* = 8), and *Drd3-Cre;Scn2a^fx/+^* mice (*n* = 6) with *Cre-GFP* in *PRC* across test days in the BM. Points represent individual mice; error bars indicate mean ± SEM. Friedman test with Dunn’s post hoc test and two-way RM ANOVA with Tukey’s post hoc test. (**F**) Left: Recording paradigm at SC-CA1. Right: I-O curve in *Drd3-Cre;Scn2a^fx/+^* mice with *Cre-GFP* in PRC and *GFP* in PRC. Inset shows representative fEPSPs. Two-way RM ANOVA with Holm-Sidak post hoc test. (**G**) Paired-pulse ratio at SC-CA1. Two-way RM ANOVA with Fisher’s LSD post hoc test. (**H**) Inset shows representative fEPSPs before (1, baseline) and after TBS (3, 50 to 60 min). Plot of average normalized fEPSP slope over time in brain slices from *Drd3-Cre;Scn2a^fx/+^* with *Cre-GFP* and *GFP* in PRC mice. (**I**) Mean normalized fEPSP slope plot at baseline, 20 to 30 min, and 50 to 60 min after TBS. Each point represents an individual brain slice; bars denote mean ± SEM.

We performed a Cre-mediated viral knockdown (*AAV9-CaMKII-Cre-GFP*) of *Scn2a* in excitatory neurons of L2/3 PRC in *Scn2a^fx/+^* mice to assess the impact on plasticity and spatial learning (Fig. 4A). Wild-type littermates (*Scn2a^+/+^*) injected with the same virus served as controls. While basal synaptic transmission was unchanged with *Scn2a* knockdown in the PRC (Fig. 4A), paired-pulse facilitation increased, indicating reduced vesicular release probability [two-way RM ANOVA: effect of condition: *P* = 0.0185; effect of interstimulus interval: *P* = 0.0293; Fisher’s least significant difference (LSD) post hoc test: ^#^(red) *P* = 0.0150 and *P* = 0.0141] (Fig. 4B). Such deficits can impair TBS-LTP. Consistent with this, knockdown of *Scn2a* in the PRC significantly impaired TBS-LTP [two-way RM ANOVA: effect of condition: *P* = 0.0014; effect of time: *P* = 0.0021; interaction: *P* = 0.0013; Tukey post hoc test: *(gray) *P* = 0.0110 and *P* = 0.0236, ^#^(black) *P* = 0.0162 and *P* = 0.0250] (Fig. 4, C and D). Impaired PRC LTP was associated with reduced spatial learning, with less than 50% of all trials completed spatially across testing days [Friedman test: *P* = 0.0053 (*Scn2a^fx/+^ Cre-GFP* in PRC), *P* = 0.0002 (*Scn2a^+/+^ Cre-GFP* in PRC); Dunn’s post hoc test: *(black) *P* = 0.0135, **(gray) *P* = 0.0020 and *P* = 0.0042] (Fig. 4E). *Scn2a^fx/+^ Cre-GFP* in PRC mice exhibited fewer spatial approach trials on test day 3 (*P* = 0.0006) and day 4 (*P* = 0.0093) compared to *Scn2a^+/+^ Cre-GFP* in PRC mice (Fig. 4E). Through PRC projections to CA1 and CA3 (*51*, *52*), a reduction in vesicular release in PRC could impair SC-CA1 TBS-LTP.

To determine whether these PRC deficits can impair TBS-induced SC-CA1 LTP, we examined whether Cre-mediated viral knockdown of *Scn2a* expression (*AAV9-CaMKII-Cre-GFP*) in the PRC could disrupt SC-CA1 TBS-LTP. Basal synaptic transmission and paired-pulse facilitation in the SC-CA1 pathway were unchanged in *Drd3-Cre;Scn2a^fx/+^* mice (Fig. 3, C and D). Cre-mediated *Scn2a* knockdown in the PRC of *Drd3-Cre;Scn2a^fx/+^* mice impaired basal synaptic transmission at the SC-CA1 synapse [two-way RM ANOVA: effect of virus: *P* = 0.0312; effect of stimulus intensity: *P* < 0.0001; interaction: *P* = 0.0004; Holm-Sidak post hoc test: ^#^(red) *P* = 0.0103, ^##^(red) *P* = 0.0020] (Fig. 4F) but left paired-pulse facilitation intact (Fig. 4G). This suggests that *Scn2a* knockdown in the PRC likely induces a postsynaptic deficit at CA1. Consistent with the deficits in basal synaptic transmission at the SC-CA1 synapse, TBS-LTP was significantly impaired [two-way RM ANOVA: effect of virus: *P* = 0.0014; effect of time: *P* < 0.0001; interaction: *P* < 0.0001; Tukey post hoc test: *(gray) *P* = 0.0130, **(gray) *P* = 0.0050 and *P* = 0.055, *(red) *P* = 0.0130, ^##^(red) *P* = 0.0068 and *P* = 0.0087] (Fig. 4, H and I).

Moreover, while *Drd3-Cre;Scn2a^fx/+^* mice exhibited normal spatial learning (Fig. 3B), Cre-mediated *Scn2a* knockdown in the PRC of *Drd3-Cre;Scn2a^fx/+^* mice impaired spatial learning (red shaded bars) (Fig. 4E). These effects were not additive, as spatial learning was disrupted to a similar extent in both *Scn2a^fx/+^* mice with Cre-mediated *Scn2a* knockdown in the PRC alone (black shaded bars) and *Drd3-Cre;Scn2a^fx/+^* mice with Cre-mediated *Scn2a* knockdown in the PRC (red shaded bars) (Fig. 4E). *Drd3-Cre;Scn2a^fx/+^ Cre-GFP* in PRC and *Scn2a^fx/+^ Cre-GFP* in PRC mice exhibited poor spatial learning compared to *Scn2a^+/+^ Cre-GFP* in PRC mice (two-way RM ANOVA: effect of condition: *P* = 0.0013, test day: *P* < 0.0001, and interaction: *P* = 0.0016). Specifically, *Drd3-Cre;Scn2a^fx/+^ Cre-GFP* in PRC mice had significantly fewer spatial approach trials on test day 2 (*P* = 0.0323), day 3 (*P* = 0.0057), and day 4 (*P* = 0.0440) compared to *Scn2a^+/+^ Cre-GFP* in PRC mice. Collectively, these findings suggest that the spatial learning deficits seen in *Scn2a^+/−^* mice stem from decreases in *Scn2a* within the PRC, specifically, rather than the hippocampus. These results further suggest that the PRC mediates spatial learning through connections to the SC-CA1 circuit.

Given that the Barnes maze task is a visuospatial task and several visual and MTL areas play a role in conveying task-relevant information to the hippocampus, we sought to rule out the possibility that reducing *Scn2a* expression in other cortical areas could impair spatial learning and SC-CA1 TBS-LTP. To address this, we reduced *Scn2a* expression via stereotaxic injection of *AAV9-CaMKII-Cre-GFP* into the anteromedial visual cortex, retrosplenial cortex, and entorhinal cortex of *Scn2a* floxed mice. In all cases, the mice with reduced *Scn2a* expression performed at comparable levels to control mice with intact *Scn2a* expression in these regions and exhibited normal levels of TBS-LTP (fig. S6). These results further suggest a degree of specificity for the PRC in regulating spatial learning and hippocampal plasticity.

### Chemogenetic excitation of cortical PNs restores spatial learning, activity levels, and PRC plasticity

*Scn2a* reduction in the *Emx1^Cre^;Scn2a^fx/+^* mice impaired spatial learning, and *Emx1^Cre^;Scn2a^fx/+^;FosEGFP* mice exhibit cell-autonomous reductions in cFos in 15 cortical brain areas, including the PRC (Fig. 2, B and C). This suggests that reductions in cortical activity are causally linked to the spatial learning deficits. To determine whether increasing cortical activity could rescue these deficits, we generated *Emx1^Cre^;hM3Dq^fx/+^;Scn2a^+/−^* mice. These mice express a modified human M3 muscarinic Gq-coupled designer receptor (*hM3Dq^fx/+^*), activated by the designer drug clozapine N-oxide (CNO) (*53*), which enables chemogenetic enhancement of depolarization and subsequent firing activity in excitatory PNs.

**Fig. 5.**
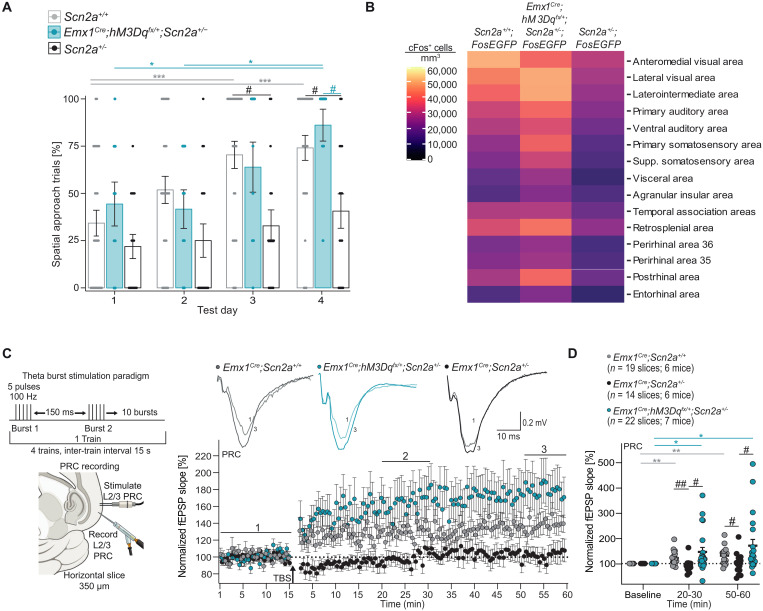
Chemogenetic excitation of cortical PNs restores spatial learning, cortical activity levels, and PRC plasticity. (**A**) Scatter bar plot showing the percentage of spatial search trials in *Emx1^Cre^;hM3Dq^fx/+^;Scn2a^+/−^* (*n* = 9), *Scn2a^+/−^* (*n* = 16), and *Scn2a^+/+^* (*n* = 27) mice across test days in the BM. Points represents individual mice; error bars indicate mean ± SEM. Friedman test with Dunn’s post hoc test and two-way RM ANOVA with Tukey’s post hoc test. Friedman test: *P = 0.0063 (*Emx1^Cre^;hM3Dq^fx/+^;Scn2a^+/−^*) and *P < 0.0001 (*Scn2a^+/+^*); ***(gray) *P* = 0.0003 and *P* = 0.0001; *(teal) *P* = 0.0487 and *P* = 0.0279; RM ANOVA: effect of genotype: *P* = 0.0388; effect of test day: *P* < 0.0001; ^#^(black) *P* = 0.0355 and *P* = 0.0490; ^#^(teal) *P* = 0.0275. (**B**) Heatmap showing the effect of chemogenetic excitation on increasing cFos-positive cell densities in the 15 brain regions of interest in *Emx1^Cre^;hM3Dq^fx/+^;Scn2a^+/−^;FosEGFP* (*n* = 4) mice when compared to either *Scn2a^+/−^;FosEGFP* (*n* = 3) or *Scn2a^+/+^;FosEGFP* (*n* = 3) mice. No statistical differences were observed in genotype comparisons between column 1 and 2, unpaired Student’s *t* test. (**C**) Left: Recording paradigm for TBS-LTP in the PRC. Right: Inset shows representative fEPSPs before (1, baseline) and after TBS (3, 50 to 60 min). Plot of average normalized fEPSP slope over time in brain slices from *Emx1^Cre^;hM3Dq^fx/+^;Scn2a^+/−^* mice treated with 1 μM CNO, *Emx1^Cre^;Scn2a^+/+^* mice, and *Emx1^Cre^;Scn2a^+/−^* mice. (**D**) Mean normalized fEPSP slope plot at baseline, 20 to 30 min, and 50 to 60 min after TBS. Each point represents an individual brain slice; bars denote mean ± SEM. Effect of genotype: *P* = 0.0218; effect of time: *P* = 0.0026; interaction: *P* = 0.0144.

We evaluated the effect of chemogenetic activation in *Emx1^Cre^;hM3Dq^fx/+^;Scn2a^+/−^* mice by administering CNO (0.25 mg/kg) daily, 30 min before Barnes maze testing. Unlike *Scn2a^+/−^* littermates, CNO-treated *Emx1^Cre^;hM3Dq^fx/+^;Scn2a^+/−^* mice exhibited normal spatial learning levels (Fig. 5A). After restoring spatial learning, we investigated the impact of chemogenetic activation on cortical activity and found that cFos immunoreactivity increased to normal levels in the 15 original cortical regions of interest (Fig. 5B). Additionally, PRC TBS-LTP was also restored with CNO treatment of brain slices from *Emx1^Cre^;hM3Dq^fx/+^;Scn2a^+/−^* mice (Fig. 5, C and D). We also confirmed that bath-applied 1 μM CNO increased spiking in L2/3 cortical PNs (*Emx1^Cre^;hM3Dq^fx/+^;Scn2a^+/+^*, before CNO: 0.6 ± 0.27 Hz, after CNO: 1.8 ± 0.54 Hz; *P* = 0.037, paired *t* test). These results suggest that chemogenetic activation of *hM3Dq* is sufficient to restore cortical activity.

### Chemogenetic activation of PRC excitatory neurons restores spatial learning and corrects deficits in PRC plasticity and SC-CA1 LTP

**Fig. 6.**
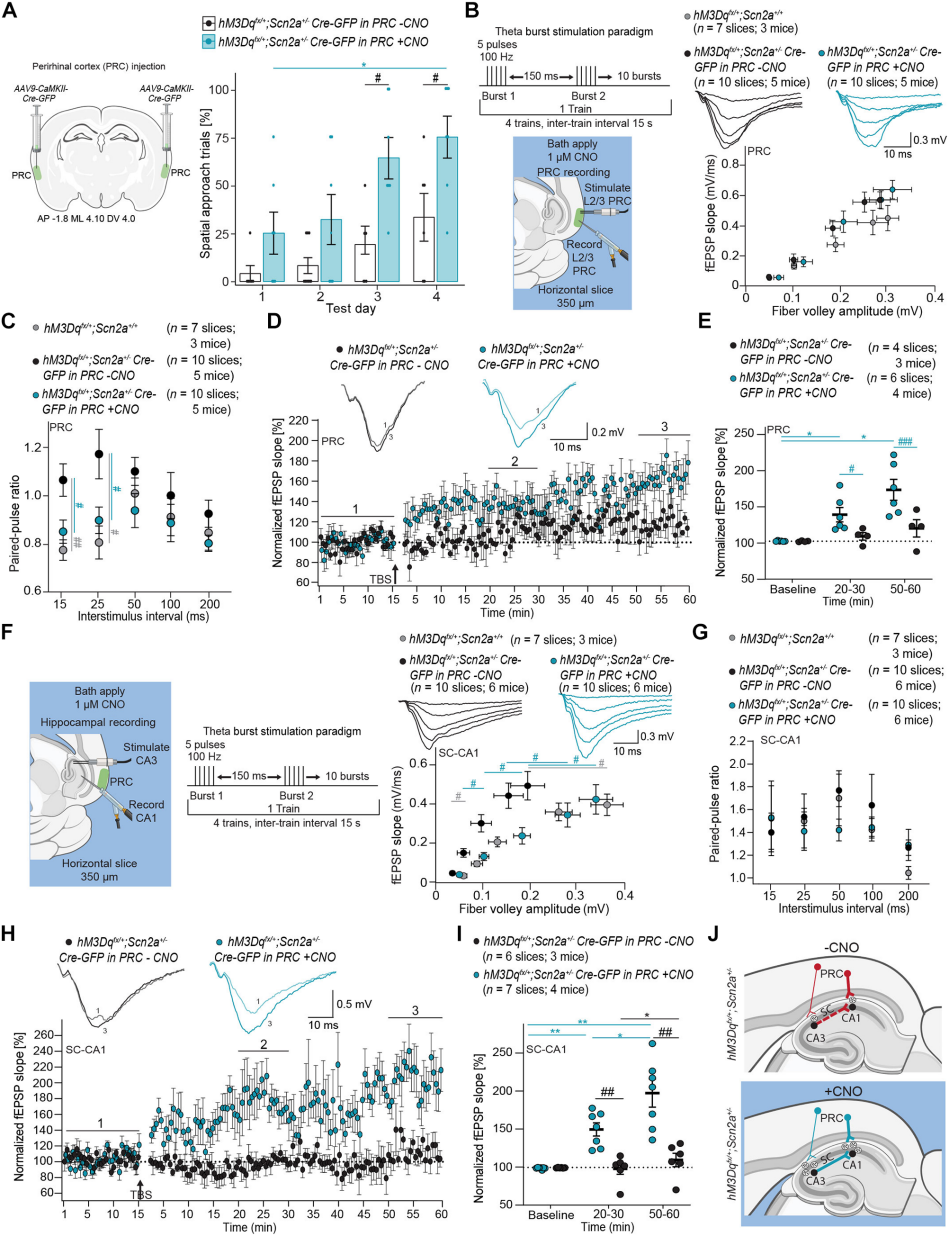
Chemogenetic activation of PRC excitatory neurons rescues spatial learning and corrects deficits in PRC and hippocampal plasticity. (**A**) Left: *AAV9-CaMKII-Cre-GFP* injection into PRC of *hM3Dq^fx/+^;Scn2a^+/−^* mice. Right: Scatter bar plot shows percent of spatial trials in *hM3Dq^fx/+^;Scn2a^+/−^* mice with *CaMKII-Cre-GFP* in the PRC treated with CNO (0.25 mg/kg) (*n* = 7) compared to untreated (*n* = 6). Friedman test with Dunn’s post hoc test and two-way RM ANOVA with Tukey post hoc test. (**B**) Left: Recording paradigm in PRC. Right: Inset shows representative fEPSPs. I-O curve for *hM3Dq^fx/+^;Scn2a^+/+^* and *hM3Dq^fx/+^;Scn2a^+/−^* with *CaMKII-Cre-GFP* in PRC with or without 1 μM CNO. Two-way RM ANOVA with Holm-Sidak post hoc test. (**C**) Paired-pulse ratio in PRC of *hM3Dq^fx/+^;Scn2a^+/+^* and *hM3Dq^fx/+^;Scn2a^+/−^* with *CaMKII-Cre-GFP* in PRC with or without CNO. Two-way RM ANOVA with Fisher’s LSD post hoc test. (**D**) Plot of average normalized fEPSP slope over time in slices from *hM3Dq^fx/+^;Scn2a^+/−^* mice with *Cre-GFP* in the PRC with or without CNO. Inset shows representative fEPSPs before (1, baseline) and after TBS (3, 50 to 60 min). (**E**) Mean normalized fEPSP slope at baseline, 20 to 30 min, and 50 to 60 min after TBS. Two-way RM ANOVA with Tukey post hoc test. (**F**) Left: Recording paradigm at SC-CA1. Right: I-O curve for *hM3Dq^fx/+^;Scn2a^+/+^* and *hM3Dq^fx/+^;Scn2a^+/−^* mice with *CaMKII-Cre-GFP* in PRC with or without CNO. Two-way RM ANOVA with Holm-Sidak post hoc test. (**G**) Paired-pulse ratio at SC-CA1 of *hM3Dq^fx/+^;Scn2a^+/+^* and *hM3Dq^fx/+^;Scn2a^+/−^* mice with *CaMKII-Cre-GFP* in PRC with or without CNO. Two-way RM ANOVA with Fisher’s LSD post hoc test. (**H**) Plot of average normalized fEPSP slope over time in *hM3Dq^fx/+^;Scn2a^+/−^* mice with *Cre-GFP* virus in the PRC with or without CNO. Inset shows representative fEPSPs before (1, baseline) and after TBS (3, 50 to 60 min). (**I**) Mean normalized fEPSP slope plot at baseline, 20 to 30 min, and 50 to 60 min after TBS. (**J**) Proposed mechanistic effect of CNO on the circuit.

To determine whether chemogenetic enhancement of PRC activity alone could rescue spatial learning and LTP deficits, we used Cre-mediated viral recombination of the *hM3Dq* floxed allele in *hM3Dq^fx/+^;Scn2a^+/−^* mice with *AAV9-CaMKII-Cre-GFP*, allowing for the expression of *hM3Dq* in PRC excitatory PNs (Fig. 6A). CNO-treated *hM3Dq^fx/+^;Scn2a^+/−^* mice with this *Cre-GFP* virus showed an increase in spatial search trials over testing days and exhibited normal levels of spatial learning, whereas CNO-untreated mice failed to navigate spatially [Friedman: *P* = 0.0056 (+CNO); Dunn’s post hoc test: *(teal) *P* = 0.0312; two-way RM ANOVA: effect of treatment: *P* = 0.0131; effect of test day: *P* < 0.0001; interaction: *P* = 0.2650; Holm-Sidak post hoc test: ^#^(day 3) *P* = 0.0201, ^#^(day 4) *P* = 0.0497] (Fig. 6A). Additionally, some *hM3Dq^fx/+^;Scn2a^+/−^* mice were inadvertently targeted in the early phases of this study to adjacent regions, including the primary somatosensory cortex, secondary somatosensory cortex, or auditory cortex. In these mistargeted cases, spatial learning behavior remained impaired at *Scn2a^+/−^* levels and was not rescued (fig. S7).

Untreated slices from *hM3Dq^fx/+^;Scn2a^+/−^* mice exhibited intact basal synaptic transmission but increased paired-pulse facilitation in the PRC, indicating abnormally reduced vesicular release probability (fig. S8, A and B). However, CNO treatment increased vesicular release probability without affecting basal synaptic transmission [two-way RM ANOVA: effect of condition: *P* = 0.0107; interstimulus interval: *P* = 0.0263; Fisher’s LSD post hoc test: ^##^(ISI 15, *hM3Dq^fx/+^*;*Scn2a^+/+^* versus *hM3Dq^fx/+^*;*Scn2a^+/−^*) *P* = 0.0031, ^#^(ISI 15, *hM3Dq^fx/+^*;*Scn2a^+/−^* with CNO versus no CNO) *P* = 0.0212, ^#^(ISI 25, *hM3Dq^fx/+^*;*Scn2a^+/−^* with CNO versus no CNO) *P* = 0.0353, ^#^(ISI 25, *hM3Dq^fx/+^*;*Scn2a^+/+^* versus *hM3Dq^fx/+^*;*Scn2a^+/−^*) *P* = 0.0105] (Fig. 6, B and C). TBS-LTP in L2/3 of the PRC was restored to wild-type levels and was significantly greater than in untreated slices, which exhibited minimal hippocampal LTP [two-way RM ANOVA: effect of treatment: *P* = 0.0223; time: *P* = 0.0011; interaction: *P* = 0.0235; Tukey post hoc test: *(teal) *P* = 0.0384 and *P* = 0.0130; Holm-Sidak post hoc test: ^#^(teal) *P* = 0.0474, ^###^(teal) *P* = 0.0006] (Fig. 6, D and E).

In the hippocampus, CNO treatment of brain slices from *hM3Dq^fx/+^;Scn2a^+/−^* mice with Cre-GFP in PRC increased basal synaptic transmission at the SC-CA1 synapse [two-way RM ANOVA: effect of condition: *P* = 0.0073; stimulus intensity: *P* < 0.0001; interaction: *P* < 0.0001; Holm-Sidak post hoc test: ^#^(gray—25 μA) *P* = 0.0496, ^#^(gray—300 μA) *P* = 0.0107, ^#^(teal—50 μA) *P* = 0.0458, ^#^(teal—100 μA) *P* = 0.0162, ^#^(teal—200 μA) *P* = 0.0185, ^#^(teal—300 μA) *P* = 0.0260] (Fig. 6F) but did not affect paired-pulse facilitation (Fig. 6G). Untreated slices showed reduced basal synaptic transmission, but paired-pulse facilitation was unaffected (fig. S8, C and D). These findings also suggest that PRC activation likely ameliorates a postsynaptic deficit at CA1 by increasing neurotransmitter release. In the absence of CNO, *hM3Dq^fx/+^;Scn2a^+/−^* replicated the effects seen in *Scn2a^+/−^* mice, exhibiting little to no hippocampal LTP, consistent with previous reports (*13*). However, CNO-treated slices exhibited hippocampal LTP comparable to wild types [two-way RM ANOVA: effect of treatment: *P* = 0.0012; time: *P* = 0.0002; interaction: *P* < 0.0001; Holm-Sidak post hoc test: ^##^(black) *P* = 0.0023 and *P* = 0.0069; Tukey post hoc test: **(teal) *P* = 0.0029 and *P* = 0.0043, *(teal) *P* = 0.0126, *(black) *P* = 0.0317] (Fig. 6, H and I). Collectively, these findings suggest that chemogenetic activation of the PRC increases release probability from excitatory neurons, enhancing basal synaptic transmission and normalizing LTP at the SC-CA1 synapse. Furthermore, chemogenetic activation of cortical circuits may offer a viable approach for ameliorating spatial learning and plasticity deficits in cases of *Scn2a* LOF mutations.

### Chemogenetic activation of the PRC restores spatial learning and plasticity deficits in the *Fmr1 KO* and *Cdkl5 KO* mouse models of ASD

**Fig. 7.**
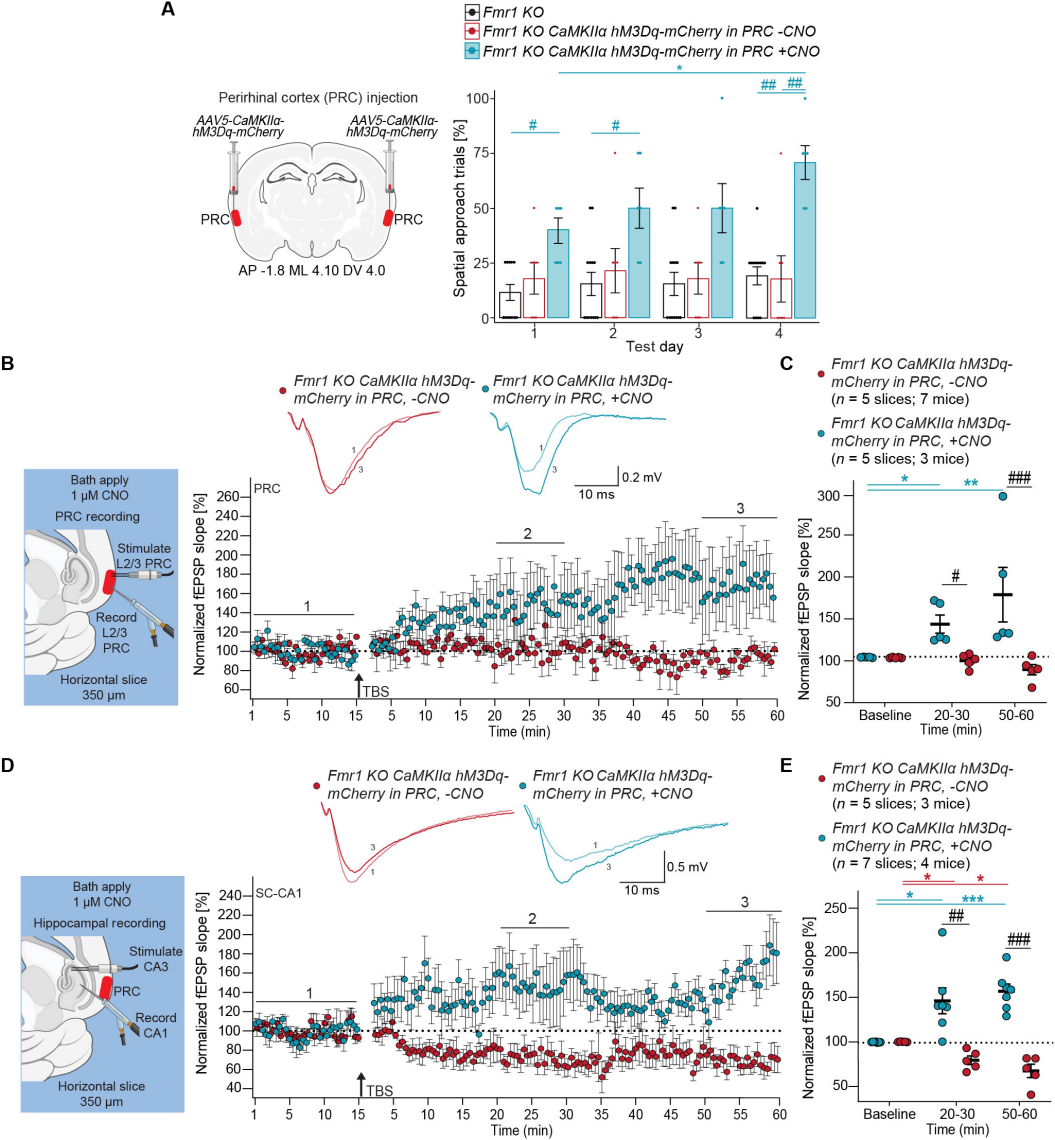
Chemogenetic activation of the PRC restores spatial learning, L2/3 PRC TBS-LTP, and SC-CA1 TBS-LTP in *Fmr1* knockout mice. (**A**) Left: Approach for *AAV5-CaMKIIa-hM3Dq-mCherry* chemogenetic excitation of excitatory neurons in the PRC of *Fmr1 KO* mice. Right: Scatter bar plot shows percentage of spatial search trials in *Fmr1 KO* mice with *CaMKIIa-hM3Dq-mCherry* in PRC with (*n* = 6) or without CNO (0.25 mg/kg) (*n* = 7). Points represent individual mice; error bars indicate mean ± SEM. Friedman test with Dunn’s post hoc test and two-way RM ANOVA with Tukey post hoc test. (**B**) Left: Recording paradigm in PRC. Right: Inset shows representative fEPSPs for each treatment, before (1, baseline) and after TBS (3, 50 to 60 min). Plot of average normalized fEPSP slope over time in brain slices from *Fmr1 KO* mice with *CaMKIIa-hM3Dq-mCherry* in the PRC with or without 1 μM CNO treatment. (**C**) Mean normalized fEPSP slope plot at baseline, 20 to 30 min, and 50 to 60 min after TBS. RM ANOVA with Tukey post hoc test. Effect of treatment: *P* = 0.0124; interaction between time and treatment: *P* = 0.0087; *(teal) *P* = 0.0437; **(teal) *P* = 0.0016; ^#^(black) *P* = 0.0474; ^###^(black) *P* = 0.0006. (**D**) Left: Recording paradigm in SC-CA1. Right: Inset shows representative fEPSPs for each treatment, before (1, baseline) and after TBS (3, 50 to 60 min). Plot of average normalized fEPSP slope over time in brain slices from *Fmr1 KO* mice with *CaMKIIa-hM3Dq-mCherry* in the PRC with or without 1 μM CNO treatment. (**E**) Mean normalized fEPSP slope plot at baseline, 20 to 30 min, and 50 to 60 min after TBS. Effect of treatment: *P* = 0.0001; interaction between time and treatment: *P* < 0.0001; *(teal) *P* = 0.0432; ***(teal) *P* = 0.0009; *(red) *P* = 0.0287 and *P* = 0.0261; ^##^(black) *P* = 0.0092; ^###^(black) *P* < 0.0001.

To explore the therapeutic effects of PRC activation on other genes linked to ASD with ID, we extended our studies to the *Fmr1 KO* and *Cdkl5 KO* mouse models. We bilaterally injected the *AAV5-CaMKII*α-*hM3Dq-mCherry* virus into PRC of *Fmr1 KO* mice (Fig. 7A), enabling chemogenetic activation of PRC excitatory PNs upon CNO treatment. In Barnes maze testing, untreated *Fmr1 KO* mice displayed impaired spatial learning (Fig. 7A), while those administered CNO (0.25 mg/kg) daily, 30 min before testing, were able to navigate spatially [Friedman test: *P* = 0.3116 (*Fmr1 KO*), *P* = 0.9420 (*Fmr1 KO* with *hM3Dq-mCherry* in the PRC, −CNO), and *P = 0.0013 (*Fmr1 KO* with *hM3Dq-mCherry* in the PRC, +CNO); *(teal) *P* = 0.0152; two-way RM ANOVA: effect of genotype: *P* = 0.0005; test day: *P* = 0.0053; interaction: *P* = 0.0430; ^#^(teal—day 1) *P* = 0.0100; ^#^(teal—day 2) *P* = 0.0305; ^##^(teal) *P* = 0.0010 and *P* = 0.0041] (Fig. 7A).

We also assessed PRC L2/3 TBS-LTP in CNO-treated and untreated slices from *Fmr1* KO mice injected with *CaMKII*α*-hM3Dq-mCherry* (Fig. 7B). Untreated slices showed negligible TBS-LTP, whereas CNO-treated slice exhibited normal levels of LTP (Fig. 7, B and C). In the hippocampus, untreated slices often displayed LTD rather than LTP at SC-CA1 following TBS (Fig. 7, D and E). In contrast, CNO-treated slices from *Fmr1* KO showed LTP at 20 to 30 min and 50 to 60 min after TBS (Fig. 7, D and E). To our knowledge, there have been no previous studies on PRC in *Fmr1-KO* mice. However, these results suggest that chemogenetically increasing PRC activity can rescue spatial learning and plasticity deficits at the PRC and SC-CA1 synapse.

**Fig. 8.**
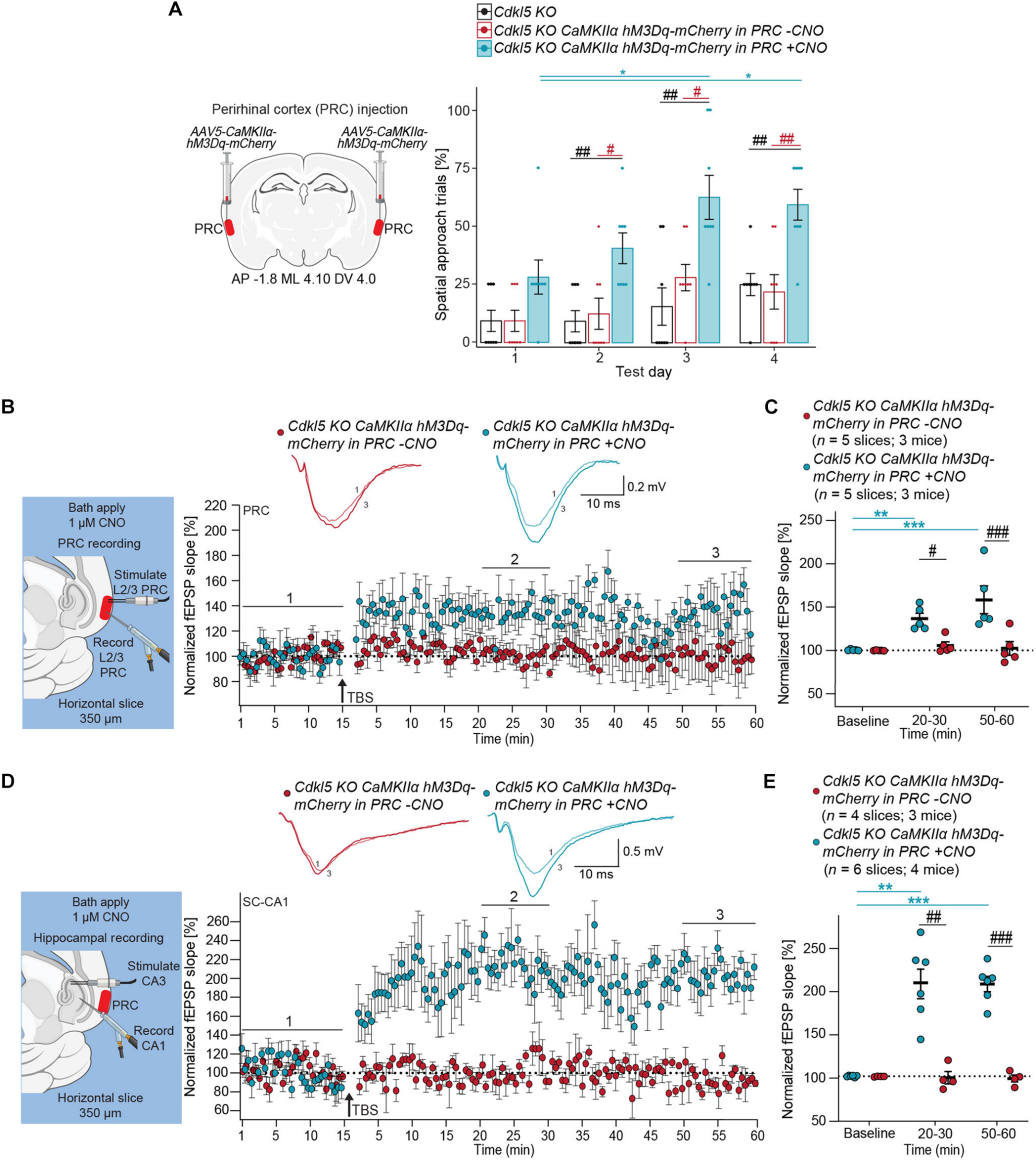
Chemogenetic activation of the PRC restores spatial learning, L2/3 PRC TBS-LTP, and SC-CA1 TBS-LTP in *Cdkl5* knockout mice. (**A**) Left: Approach for *AAV5-CaMKIIa-hM3Dq-mCherry* chemogenetic excitation of excitatory PNs in the PRC of *Cdkl5 KO* mice. Right: Scatter bar plot shows the percentage of spatial search trials in *Cdkl5 KO* mice with *CaMKIIa-hM3Dq-mCherry* in the PRC receiving no CNO (*n* = 8) and CNO (0.25 mg/kg) daily, 30 min before testing (*n* = 8) across test days in the BM. Points represent individual mice; error bars indicate mean ± SEM. Friedman test with Dunn’s post hoc test and two-way RM ANOVA with Holm-Sidak post hoc test. (**B**) Left: Recording paradigm in PRC. Right: Inset shows representative fEPSPs for each treatment. Plot of average normalized fEPSP slope over time in brain slices from *Cdkl5 KO* mice with *hM3Dq-mCherry* in the PRC with and without CNO. (**C**) Mean normalized fEPSP slope plot at baseline, 20 to 30 min, and 50 to 60 min after TBS. Two-way RM ANOVA with Tukey post hoc test. Effect of treatment: *P* = 0.0053; effect of time: *P* = 0.0016; interaction: *P* = 0.0040; **(teal) *P* = 0.0060; ***(teal) *P* = 0.0003; ^#^(black) *P* = 0.0305; ^###^(black) *P* = 0.0001. (**D**) Left: Recording paradigm for SC-CA1. Right: Inset shows representative fEPSPs for each condition. Plot of average normalized fEPSP slope over time in brain slices from *Cdkl5 KO* mice with *hM3Dq-mCherry* in the PRC with and without CNO. (**E**) Mean normalized fEPSP slope plot at baseline, 20 to 30 min, and 50 to 60 min after TBS. Two-way RM ANOVA with Tukey post hoc test. Effect of treatment: *P* = 0.0001; effect of time: *P* = 0.0005; interaction: *P* < 0.0001; **(teal) *P* = 0.0063; ***(teal) *P* = 0.0002; ^##^(black) *P* = 0.0034; ^###^(black) *P* = 0.0001.

We applied the same strategy to *Cdkl5 KO* mice by bilaterally injecting the *AAV5-CaMKII*α*-hM3Dq-mCherry* virus into PRC (Fig. 8A), enabling chemogenetic activation of PRC excitatory PNs upon CNO treatment. Untreated *Cdkl5* KO mice displayed spatial learning deficits in the Barnes maze (Fig. 8A), while those given CNO (0.25 mg/kg) daily, 30 min before testing, were able to navigate spatially [Friedman test: *P* = 0.1671 (*Cdkl5 KO*), *P* = 0.0803 (*Cdkl5 KO CaMKIIa-hM3Dq-mCherry* in the PRC −CNO), and *P = 0.0122 (*Cdkl5 KO CaMKIIa-hM3Dq-mCherry* in the PRC +CNO); Dunn’s post hoc test: *(teal) *P* = 0.0490 and *P* = 0.0131, *(red) *P* = 0.0480; two-way RM ANOVA: effect of genotype: *P* < 0.0001; test day: *P* = 0.0001; interaction: *P* = 0.3073; Holm-Sidak post hoc test: ^#^(red) *P* = 0.0243 and *P* = 0.0234, ^##^(red) *P* = 0.0053, ^##^(black) *P* = 0.0052, *P* = 0.0057, and *P* = 0.0027] (Fig. 8A). PRC L2/3 TBS-LTP in CNO-untreated slices from *Cdkl5 KO* mice showed negligible TBS-LTP, whereas CNO-treated slice exhibited normal LTP levels (Fig. 8, B and C). These plasticity deficits are consistent with previous reports of reduced PRC function in *Cdkl5 KO* mice (*54*, *55*). In the hippocampus, untreated slices from *Cdkl5 KO* mice exhibited negligible SC-CA1 LTP (Fig. 8, D and E). Conversely, CNO-treated slices exhibited moderate TBS-LTP at 20 to 30 min after TBS, which increased significantly by 50 to 60 min after TBS (Fig. 8, D and E). These findings indicate that chemogenetic enhancement of PRC activity can restore spatial learning and both PRC and hippocampal plasticity deficits in *Cdkl5 KO* mice. Collectively, in three genetically diverse ASD mouse models, our results demonstrate that activation of PRC excitatory neurons can improve learning impairments.

## DISCUSSION

Our findings in *Scn2a^+/−^* mice show that reduced *Scn2a* leads to decreased cFos activity in the cortex, impairing spatial learning. This reduction in cortical excitability may reflect initial roles for *Na_v_1.2* in action potential initiation and propagation in young neurons and later roles in somatodendritic excitability and action potential backpropagation in mature neurons (*31*–*33*). In contrast, CA1 PNs, which predominantly express *Na_v_1.6*, and *Na_v_1.1*-expressing interneurons are not functionally impaired with reductions in *Scn2a* (*31*, *56*–*58*). This may explain why cFos changes are observed in the cortex but not in the hippocampus.

Previous research suggested that impaired hippocampal LTP drives spatial learning deficits in *Scn2a^+/−^* mice (*13*). While *Drd3-Cre;Scn2a^fx/+^* mice with reduced *Scn2a* across the hippocampus showed intact spatial learning and LTP, reducing *Scn2a* in the PRC of these mice disrupted spatial navigation. This indicates that PRC hypoactivity is a primary cause of spatial learning deficits. *Scn2a* reduction may impair neuronal excitability and LTP by affecting *Na_v_1.2*-dependent back-propagating spikes, a key source of dendritic depolarization in mature cortical neurons (*31*, *59*). Spratt *et al*. (*31*) demonstrated that reduced *Scn2a* impairs LTP in L5 PNs but did not show cognitive impairment. However, *Rbp4-Cre^+/−^;Scn2a^fx/+^* and *Ntsr1-Cre;Scn2a^fx/+^* mice, with *Scn2a* reductions in cortical L5 and L6, did not exhibit spatial learning deficits, while reductions in *Scn2a* encompassing the upper cortical layers did cause behavioral deficits. In *hM3Dq^fx/+^;Scn2a^+/−^* mice, *hM3Dq* activation by CNO enhanced PRC synaptic release probability, increased PRC LTP, normalized SC-CA1 I-O dynamics, and rescued LTP and spatial learning deficits. In *Fmr1* and *Cdkl5* KO mice, similar effects were observed, with chemogenetic activation of PRC excitatory PNs, normalizing PRC LTP, SC-CA1 LTP, and spatial learning deficits.

While the hippocampus is central to spatial navigation due to its place cells, spatially tuned neurons are also present in cortical areas like the entorhinal and primary somatosensory cortices (*60*–*63*). Place cells exhibit heightened firing in specific locations (place fields) to encode spatial position. The PRC, with its reciprocal connections to these cortical areas and projections to the hippocampus, could influence place cell activity and spatial learning (*64*–*66*). Place cells maintain stable place fields in familiar environments (*67*, *68*), and bilateral PRC lesions can disrupt place cell stability (*50*), underscoring its role in spatial memory integration. Additionally, the PRC integrates space-object information to encode the “spatial scene,” and strongly phase-locks to hippocampal theta (*49*), which might modulate hippocampal place cell activity and affect SC-CA1 plasticity.

The PRC is also crucial for familiarity detection, which involves recognizing previously encountered stimuli without recalling contextual details (*69*, *70*). In tasks like the Barnes maze, intact familiarity detection is essential for spatial accuracy. Our findings in the *Scn2a^+/−^*, *Fmr1*, and *Cdkl5* KO mice suggest that PRC hypoactivity impairs hippocampal function, causing spatial learning deficits. Current literature indicates monosynaptic PRC projections to CA1 (*51*, *71*) and CA3 (*52*) in addition to polysynaptic pathways from PRC → lateral entorhinal L2/3 → CA1 and PRC → postrhinal → hippocampus (*51*, *64*, *72*, *73*). Since *Scn2a^+/−^*, *Fmr1* KO, and *Cdkl5 KO* neurons at SC-CA1 show normal paired-pulse facilitation, indicative of unaltered presynaptic release (*13*, *16*, *41*), our model suggests that PRC hypoactivity impairs activation most likely at CA1, affecting TBS-LTP. Thus, it is possible that PRC connections to the hippocampus facilitating SC-CA1 TBS-LTP encode the familiarity of spatial routes. PRC projection to the postrhinal and entorhinal cortices, and their interconnections with other MTL structures, potentially explains the widespread cortical hypoactivity in the cFos datasets and the co-occurrence of deficits in hippocampal LTP and place cell dynamics (*12*, *13*, *74*–*76*) in the *Scn2a^+/−^* and *Fmr1 KO* mice.

Our findings in *Scn2a^+/−^*, *Fmr1 KO*, and *Cdkl5 KO* mice suggest the PRC as a potential target for noninvasive neurological interventions to improve cognitive difficulties associated with ASD. Previous imaging studies have shown PRC hypoactivity in children with ASD (*77*) and impaired PRC function in *Cdkl5* KO mice (*54*, *55*). To our knowledge, PRC function had not been previously investigated in *Fmr1 KO* or *Scn2a^+/−^* mice. Targeted transcranial magnetic stimulation (TMS) or transcranial focused ultrasound could potentially replicate the effects of chemogenetic approaches, enhancing neuronal activity and LTP within the PRC. Both approaches have been used to transiently alter cortical processing and enhance brain excitability and plasticity (*78*–*82*). Overall, our findings reveal shared neurobiological underpinnings of spatial learning deficits in three different ASD mouse models, offering a therapeutic target for treating learning impairments.

## MATERIALS AND METHODS

### Experimental design

#### Animal subjects

All procedures were approved by the National Institutes of Health (NIH)/National Institute on Alcohol Abuse and Alcoholism (NIAAA) Animal Care and Use Committee and were in accordance with the regulations outlined in the *Guide for Care and Use of Laboratory Animals* by the National Research Council (protocol #LIN-MA-1, animal assurance number A4149-01). All mice were bred in the Fishers Lane Animal Center, which is accredited by the Association for Assessment and Accreditation of Laboratory Animal Care International. Mice were housed under standard conditions with ad libitum access to food and water, under a 12-hour light/dark cycle. Both male and female mice were used in this study, and any sex-specific effects on results are detailed in the supplementary figures.

Mice were obtained from the Jackson Laboratory (JAX) or the Mutant Mouse Resource and Research Center (MMRRC) and bred onsite: C57BL/6J mice (JAX 000664), *Scn2a^fx/+^* mice (JAX 035553), *Emx1^Cre^* mice (JAX 005628), *Drd3-Cre* mice (MMRRC 034610-UC), *Rbp4-Cre* mice (MMRRC 037128-UCD), *Ntsr1-Cre* mice (JAX 033365), *R26-LSL-hM3Dq-DREADD* mice (JAX 026220), *Ai14D* mice (JAX 007914), *FosEGFP* mice (JAX 014135), *Fmr1 KO* mice (JAX 004624), and *Cdkl5 KO* mice (JAX 021967). *Scn2a^+/−^* mice (*13*) were provided by K. Bender from the University of California, San Francisco. Mice were generated from in-house crosses and were genotyped commercially by Transnetyx (Memphis, TN). In-house crosses were as follows: *Emx1^Cre^;Scn2a^fx/+^*, *Emx1^Cre^;Scn2a^fx/+^;TdTom*, *Drd3-Cre;Scn2a^fx/+^*, *Drd3-Cre;Scn2a^fx/+^;TdTom*, *Rbp4-Cre;Scn2a^fx/+^*, *Rbp4-Cre;Scn2a^fx/+^;TdTom*, *Ntsr1-Cre;Scn2a^fx/+^*, *Ntsr1-Cre;Scn2a^fx/+^;TdTom*, *Emx1^Cre^;hM3Dq^fx/+^;Scn2a^+/−^*, *hM3Dq^fx/+^;Scn2a^+/−^*, and *Emx1^Cre^;hM3Dq^fx/+^;Scn2a^+/−^;Fos-EGFP^+/−^*, with associated littermate controls used from these crosses also.

### Barnes maze behavioral assay

To evaluate spatial navigation behavior, mice underwent a Barnes maze task. The Barnes maze comprises a circular platform with a diameter of 91 cm, elevated 78 cm above the floor (68010, Stoelting). The platform is equipped with 20 evenly spaced holes, measuring 6.35 cm by 6.35 cm. Nineteen of these holes are shallow (2 cm deep), while one, designated as the “escape hole,” contains a deeper container attachment (5 cm deep, 25.5 cm long). Surrounding the maze are four floor lamps, each arranged in distinct configurations to brightly illuminate the maze. Two of the floor lamps emitted 75-W red light, while the other two used 72-W bright white light. Animals were acclimated to the experimental room for 1 hour before trial start. Mice were initially introduced to the maze for a 5-min habituation trial and then guided to the escape hole to ensure awareness of its location. Mice receiving injections were intraperitoneally injected with either saline or CNO (0.25 mg/kg) (Biotechne Tocris, #6329) following the habituation trial. After 30 min, mice were subjected to acquisition trials over 4 days, with four 3-min trials each day and an intertrial interval of 30 min. If the escape compartment was not entered within 3 min, mice were guided to the escape hole. In addition to the naturally aversive stimulus of the bright light from the floor lamps on the maze, an aversive, high-decibel noise stimulus (white noise, 70 dB) was played after 30 s of the 3-min trial. The noise stimulus ended upon exit through the escape hole. In surgically naïve mice, behavior tests were conducted at postnatal day 20 to postnatal day 24. In mice that underwent surgical procedures, tests were conducted from postnatal day 34 to postnatal day 50.

For behavioral analysis, mice were tracked using AnyMaze software (#60605, Stoelting), and trials were categorized as either “spatial search” or “serial search.” The pathway analysis criteria for serial and spatial learning described in Fig. 1A were determined a priori after several months of analyzing behavior patterns in wild-type mice on the Barnes maze. During the initial one to two trials on the first day of testing, mice typically engage in random searches, crossing the maze haphazardly to find the escape hole (Fig. 1A). Serial searches involve inspecting each hole within two or more quadrants, while spatial searches are characterized by a focus on the quadrant containing the escape hole or a direct approach to it (Fig. 1A). To define spatial behavior, a limit of three or fewer erroneous selections was established.

At the start of the task, mice are placed in the center of the maze with a cover temporarily secured to confine them. Once the cover is removed, mice often dart into a hole, possibly perceiving it as a safe starting point, before proceeding to select holes in search of the escape route. Across test days (days 1 to 4), we observed that the hole initially chosen by the mice tended to shift progressively closer to the escape quadrant. To account for this specific behavioral pattern, an additional error selection (i) was incorporated into the analysis. Therefore, spatial searches are defined as those with three or fewer erroneous selections.

To ensure accuracy of the software, a researcher simultaneously manually scored the trials. Serial searches involve mice inspecting each hole within two or more quadrants to locate the escape hole, while spatial searches mainly focus on the quadrant containing the escape hole or move directly to it. The proportion of spatial and nonspatial trials was quantified after each day of testing based on trial performance. The number of mice subjected to the Barnes maze represents *n*, with specific numbers indicated in figure captions.

### Cre recombination cell count quantification

Quantification was conducted using Imaris software (Oxford Instruments, version 10.0.0). Brain regions of interest were demarcated by a 300 μm × 1000 μm area, identified based on anatomical location (e.g., PRC dorsal to the rhinal sulcus at plate 58 of the Allen Brain Atlas). Using the Imaris software, cells were counted using spot detection (corresponding to TdTomato-labeled neurons) selectively within the regions of interest. The following parameters were applied: spot diameter set at 10 μm (matching the smallest cell diameter aimed for detection), and the threshold was manually adjusted to optimize the signal-to-noise ratio by choosing the “quality” option. The resulting total cell count was displayed in the statistics tab, and this value was used for data analysis and qualitative measurement match (−, +, or ++) of expression (Table 2). Each experiment was conducted in triplicate, with *n* representing each assessed brain.

### Light-sheet imaging

Mice aged postnatal day 21 to postnatal day 24 received injections of either saline or CNO (0.25 mg/kg) 30 min before exposure to a novel open field. After 1 hour, mice were anesthetized with pentobarbital (50 mg/kg) and then transcardially perfused with phosphate-buffered saline (PBS; pH 7.4) followed by 4% paraformaldehyde (PFA). After overnight shaking in 4% PFA at 4°C, brains were washed with 1× PBS and then transferred to 0.02% sodium azide/1× PBS solution. LifeCanvas Technologies provided light-sheet images of the entire brain and cell density counts. Antibodies used were goat green fluorescent protein (GFP) antibody (#GPCA-GFP, EnCor Biotechnology) and rabbit NeuN antibody (#24307, Cell Signaling Technology). Data contained the identified region (verified via Mouse Brain Atlas overlay), cell count, volume (in mm^3^) of the region, cell density (cells per mm^3^), and the genotype of the animal. Cells were analyzed in three channels: coexpression of cFos and NeuN, cFos alone, and NeuN alone. Cell density averages were evaluated per brain region across genotypes using an in-house developed R pipeline (GitHub link). Layer 1 of the cortex was excluded from analysis due to high levels of autofluorescence resulting from light scattering. Heatmaps were generated with average cell density values in brain regions that showed significant differences between genotypes. Significance was set at *P* < 0.05. Regions with cell counts of zero were omitted from the analysis. Each experiment was conducted in triplicate, with *n* representing each assessed brain.

### Slice preparation

Using standard methods, horizontal brain sections (350 μm) containing hippocampus and PRC (*54*) were collected from mice aged postnatal day 21 (surgically naïve mice) to postnatal day 70 (mice that underwent viral injection and behavior testing). For slice preparation from surgically naïve mice, the cutting solution contained 85 mM NaCl, 2.5 mM KCl, 1.25 mM NaH_2_PO_4_-H_2_O, 3 mM MgSO_4_·7H_2_O, 0.5 mM l-ascorbic acid, 0.5 mM CaCl_2_, 75 mM sucrose, 25 mM d-(+)-glucose, and 25 mM NaHCO_3_. For slice preparation from mice that underwent viral injection, the cutting solution contained 2.5 mM KCl, 1.25 mM NaH_2_PO_4_-H_2_O, 0.4 mM l-ascorbic acid, 3 mM MgSO_4_·7H_2_O, 200 mM sucrose, 20 mM d-(+)-glucose, and 26 mM NaHCO_3_. Both solutions were neutral pH (i.e., 7.3), 310 mOsm, and saturated with 95% O_2_ and 5% CO_2_. Once cut, slices were transferred to an incubation chamber (Campden Instruments, model 7470) filled with oxygenated artificial cerebrospinal fluid (ACSF) (standard Ringer’s solution, 300 mOsm: 119 mM NaCl, 1.3 mM MgSO_4_-7H_2_O, 2.5 mM KCl, 1 mM NaH_2_PO_4_-H_2_O, 2.5 mM CaCl_2_, 11 mM d-(+)-glucose, and 26.2 mM NaHCO_3_, pH 7.4) and then incubated at 32°C for 30 min. Slices were kept at room temperature for 30 min before transfer to the recording chamber (30°C oxygenated ACSF, 2 to 3 ml/min). Heating was maintained with LinLab 2 (Scientifica, version 1.0.19.50).

### Field potential recording electrophysiology

Recordings used bipolar electrodes for stimulation and 2- to 6-megohm micropipettes containing ACSF for recording. For PRC, the stimulating electrode was positioned into L2/3 on the temporal side of the rhinal sulcus, while the recording electrode was placed in the same layer 400 μm away in the rostral direction (*54*). For hippocampal recordings, stimulation was applied to SCs from area CA3, and stratum radiatum in area CA1 was recorded. A stimulus generator (model DS3, Digitimer Ltd.) delivered constant-current square pulses (0.1 ms, 25 to 300 μA), and signals were low pass–filtered at 1 kHz. Parameters were recorded and analyzed using the Clampex suite (Molecular Devices, version 11.1). fEPSPs were recorded every 10 s. I-O curves were determined by measuring the fiber volley (FV) and fEPSP amplitudes at 25, 50, 100, 200, and 300 μA for 10 sweeps. Stimulus intensity was adjusted to induce a half-maximal fEPSP amplitude. Paired-pulse ratios were determined by calculating the ratio of the amplitude of the test EPSP (second response) to the conditioning EPSP (first response) at interstimulus intervals of 15, 25, 50, 100, and 200 ms. For LTP, a baseline was established by recording responses every 30 s for 15 min before applying TBS (four trains every 15 s, each train comprising 10 bursts of five pulses at 100 Hz, interburst interval 150 ms). After TBS, responses were recorded every 30 s for 60 min. For studies involving *hM3Dq*, 1 μM CNO was bath-applied to activate this designer receptor. FV amplitude and fEPSP slope were compared using a two-way RM ANOVA followed by a Tukey post hoc test across genotypes and stimulus intensities to derive I-O curves. A two-way RM ANOVA with a Fisher’s LSD post hoc test was used to assess the effect of genotype and interstimulus interval on the paired-pulse ratio. Baseline and post-LTP induction responses across genotypes and time were also compared by RM ANOVA. As reported in figure captions, *n* represents the number of slices recorded, followed by the number of animals used. Significance was defined as *P* < 0.05, with α = 0.05.

### Stereotaxic viral injections

Juvenile mice (P21 to P24) were anesthetized with 2 to 3% isoflurane using precision vaporizers (Patterson Scientific, Waukesha, WI). A 1 to 2% solution of lidocaine was used as a topical gel for instillation into the external ear canals before placement in the motorized stereotaxic system fitted with a computer-controlled drill (Neurostar, Kopf Instruments, Germany). During surgery, lubricant eye ointment was applied, and body temperature was maintained at 37.0°C with a heating pad. After incising the scalp to expose the skull, connective tissues were cleaned off using 3% hydrogen peroxide. A small hole was drilled (0.7 mm diameter), and virus was injected into target regions using a Hamilton syringe (catalog no. 7635-01) with a 32-gauge needle (catalog no. 7803-04) at a rate of 0.05 μl/min for a total volume of 0.2 μl. After injection, the syringe was left in place for 5 min before being slowly withdrawn.

Stereotaxic coordinates were determined relative to bregma: Retrosplenial cortex: AP (anteroposterior): −2.06 mm, ML (mediolateral): 0.17 mm, DV (dorsoventral): 0.63 mm; PRC: AP: −1.8 mm, ML: ±4.1 mm, DV: −4.0 mm; anteromedial visual area: AP: −2.54, ML: 1.75, DV: 0.55; entorhinal cortex: AP: −4.36, ML: 3.9, DV: 1.4. After injection, mice were incubated for at least 2 weeks before undergoing behavioral testing as required. *Scn2a^fx/+^*, *Drd3-Cre*;*Scn2a^fx/+^*, and *hM3Dq^fx/+^;Scn2a^+/−^* mice, along with their wild-type littermates, were injected with *CaMKII-GFP-Cre* virus (Addgene, plasmid #105551, pENN.AAV.CamKII.HI.GFP-Cre.WPRE.SV40) into the targeted brain area. *Fmr1* KO and *Cdkl5* KO mice were injected with *CaMKII*α*-hM3Dq-mCherry* virus [Addgene, plasmid #50476, pAAV-CaMKIIa-hM3D(Gq)-mCherry (AAV5)] into the PRC. After behavior tests, mice were randomly selected for imaging to confirm virus injection sites or for electrophysiological recordings. Slices collected for electrophysiology from virus-injected mice were anatomically confirmed for injection sites upon placement into the recording chamber.

### Imaging

Mice were anesthetized and transcardially perfused using standard methods. After overnight shaking in 4% PFA at 4°C, brains were washed with 1× PBS and then placed in 30% sucrose for cryoprotection. Brains were then embedded in optimal cutting temperature (OCT) compound, frozen using dry ice, and stored at −80°C until sectioning. Tissue sections were cut at 30 μm using a cryostat, mounted onto slides, and counterstained with DAPI (4′,6-diamidino-2-phenylindole) Flouromount G (0100-20, Southern Biotech, Birmingham, AL). The slides were coverslipped and stored at 4°C until imaging. Slices were captured using a Keyence BZ-X810 microscope (BZ-X800 Viewer, version 1.1.1.8) with specific BZ-X filters [DAPI: excitation wavelength 360/40 nm (OP-87762, Keyence), GFP: excitation wavelength 470/40 nm (OP-87763, Keyence), and tetramethyl rhodamine isothiocyanate (TRITC): excitation wavelength 545/25 nm (OP-87764, Keyence)] at ×2 magnification to confirm injection sites and TdTomato expression. Regions were then imaged at 10× for quantification.

### Statistics

Statistical analyses were conducted using Prism 9.0 (GraphPad) or R version 4.1.0 with the environment RStudio version 2022.07.1. For experiments, at least three mice from a minimum of three separate litters were used. Non-Gaussian data were either log-transformed for parametric testing or subjected to nonparametric tests, as specified in Results and figure legends. Significance was consistently reported at α = 0.05. Details on the statistical tests used are provided in the figure captions, as well as within each appropriate section of the experimental design.
