# Supplementary material for: Perirhinal cortex abnormalities impair hippocampal plasticity and learning in Scn2a, Fmr1, and Cdkl5 autism mouse models
Source: Sci Adv. 2025 Mar 7;11(10):eadt0780. doi: 10.1126/sciadv.adt0780 (PMC11887805; doi:10.1126/sciadv.adt0780)
Supplement: Supplementary file 1 — Figs. S1 to S8 [file sciadv.adt0780_sm.pdf]

Science Advances
AAAS

Supplementary Materials for

Perirhinal cortex abnormalities impair hippocampal plasticity and learning in *Scn2a*, *Fmr1*, and *Cdkl5* autism mouse models

Rachel E. Keith *et al.*

Corresponding author: Michelle W. Antoine, michelle.antoine@nih.gov

Sci. Adv. **11**, eadt0780 (2025)
DOI: 10.1126/sciadv.adt0780

This PDF file includes:

Figs. S1 to S8

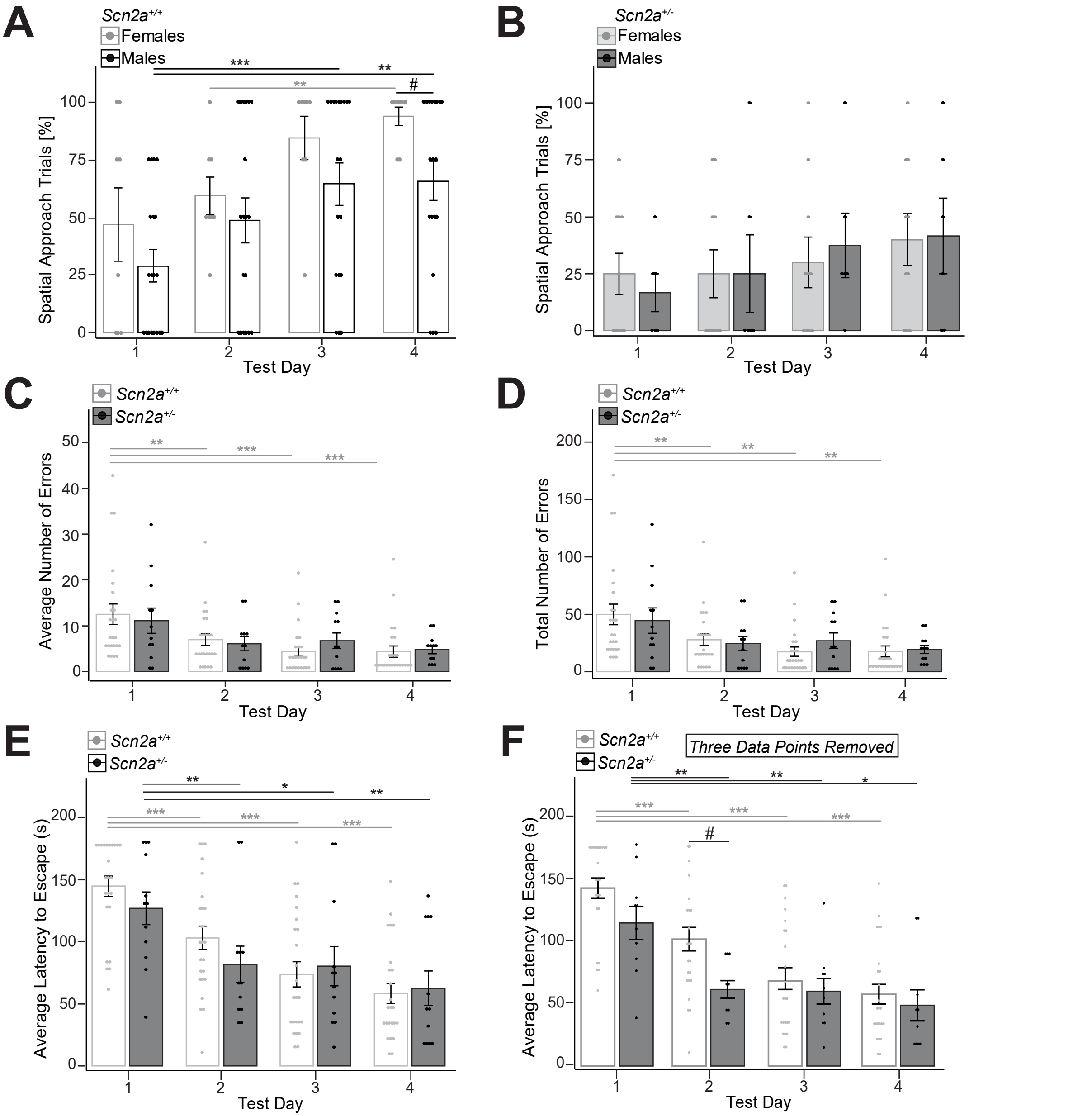

Supplemental Fig. 1: No sex differences in spatial learning observed across genotypes.

(A) Scatter bar plot with spatial search trials observed in *Scn2a*$^{+/+}$ females (n = 8) and males (n = 19) across test days in the BM. Friedman test with Dunn's post-hoc test: p = 0.0131 (Females), and p < 0.0001 (Males). ***(black) p = 0.0008. **(black) p = 0.0026. **(gray) p = 0.0051. Two-way RM ANOVA with a Tukey post-hoc test: No effect of sex: p = 0.1356; test day: p < 0.0001; ***(gray) p = 0.0003, ***(black) p = 0.0007 and p < 0.0001, respectively. #(black) p = 0.0285.

(B) Same as **(A)** for *Scn2a*$^{+/-}$ mice. *Scn2a*$^{+/-}$ females (n = 10) and males (n = 6). Friedman: p = 0.5017 (Females), and p = 0.5471 (Males). RM ANOVA: No statistical differences.

(C) Scatter bar plot of average errors per test day for *Scn2a*$^{+/+}$ (n = 27) and *Scn2a*$^{+/-}$ mice (n = 16) in the BM. RM-ANOVA with Tukey post-hoc test. Data also analyzed in (**D**) and (**E**). Effect of test day: p < 0.0001; **(gray) p = 0.0047. ***(gray) p = 0.0007 and p = 0.0005, respectively.

(D) Same as (**C**) for the total number of errors per test day for mice. Effect of test day: p < 0.0001; **(gray) p = 0.0047. ***(gray) p = 0.0007 and p = 0.0005, respectively.

(E), Same as (**C**) for average latency to enter the escape hole. Effect of test day: p < 0.0001; ***(gray) p = 0.0002, p < 0.0001 and p < 0.0001, respectively; **(black) p = 0.0081 and p = 0.0032, respectively. *(black) p = 0.0111.

(F), Same as (**E**) but two *Scn2a*$^{+/-}$ mice and one *Scn2a*$^{+/+}$ mouse which fail to escape on day 2 and 3 are removed. No effect of genotype: p = 0.1075; effect of test day: p < 0.0001. ***(gray) p = 0.0002, p < 0.0001, and p < 0.0001, respectively; **(black) p = 0.0049 and p = 0.0080, respectively; *(black) p = 0.0117. #p = 0.0130.

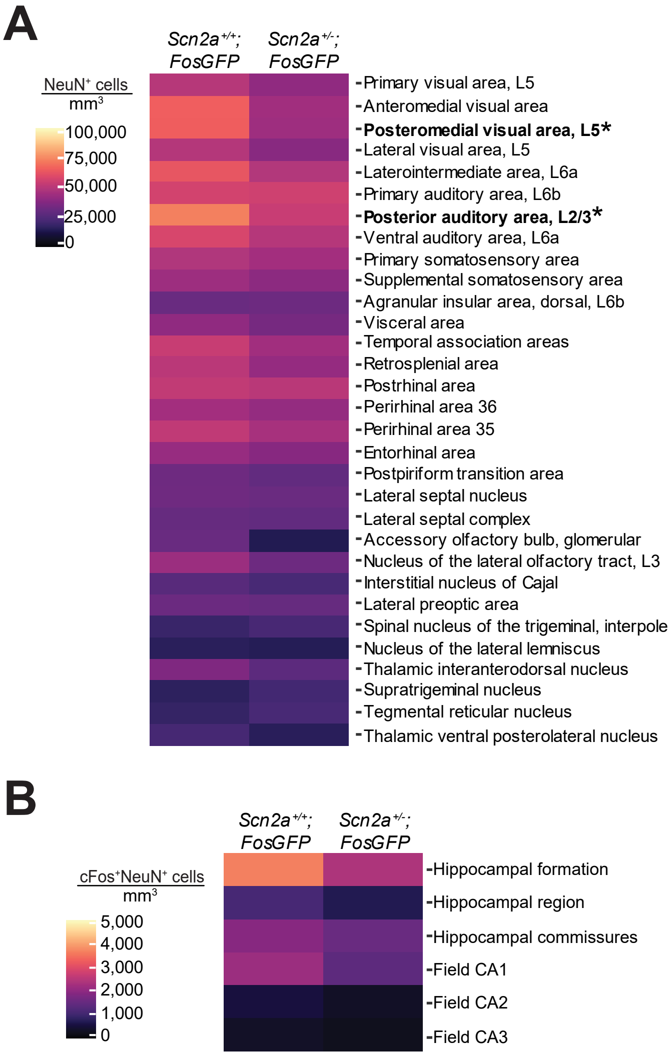

Supplemental Fig. 2: Comparable neuronal densities between *Scn2a$^{+/-}$;FosGFP* and *Scn2a$^{+/+}$;FosGFP* mice, with no differences in hippocampal cFos-positive neurons.

(A) Heatmap of neuronal densities across 31 brain regions of interest. Statistical differences found in posteromedial visual area, L5 ($p = 0.040$) and posterior auditory area, L2/3 ($p = 0.044$).

(B) Heatmap of cFos-positive neuronal densities in the hippocampus, showing no genotype differences. Statistics were computed at $\alpha = 0.05$ with unpaired two-tailed t-tests.

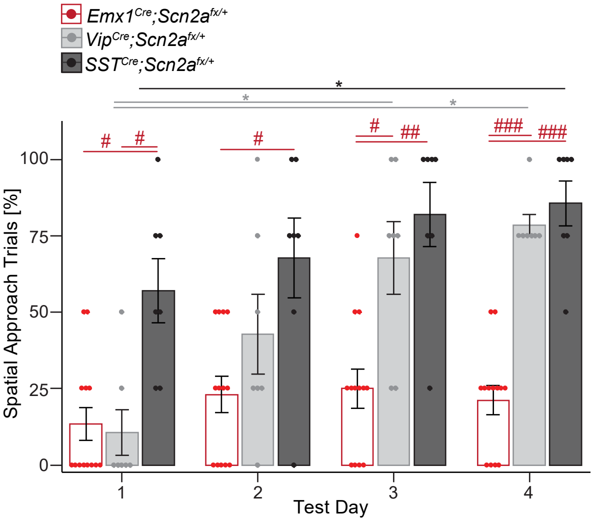

Supplemental Fig. 3: Reduced *Scn2a* expression in SST or VIP interneurons does not cause spatial learning impairments. Scatter bar plot of spatial search trials in *Emx1*^{Cre}*;Scn2a*$^{fx/+}$ (n = 13), *Vip*^{Cre}*;Scn2a*$^{fx/+}$ mice (n = 7) and *SST*^{Cre}*;Scn2a*$^{fx/+}$ mice (n = 7) across test days in the BM. Points represents individual mice; error bars indicate mean ± SEM. Friedman test with Dunn's post-hoc test: p = 0.4914 (*Emx1*^{Cre}*;Scn2a*$^{fx/+}$), p = 0.0030 (*Vip*^{Cre}*;Scn2a*$^{fx/+}$), and p = 0.0562 (*SST*^{Cre}*;Scn2a*$^{fx/+}$). *(black) p = 0.0452. *(gray) p = 0.0312 and p = 0.0114, respectively. Two-way RM ANOVA with Holms-Sidak post-hoc test: Effect of genotype: p < 0.0001; testing day: p < 0.0001; interaction between genotype and testing day: p = 0.0011. ### p < 0.0001, ## p = 0.0021. #(Day 1) p = 0.0109 and p = 0.0118, respectively. #(Day 2) p = 0.0314. #(Day 3) p = 0.0252.

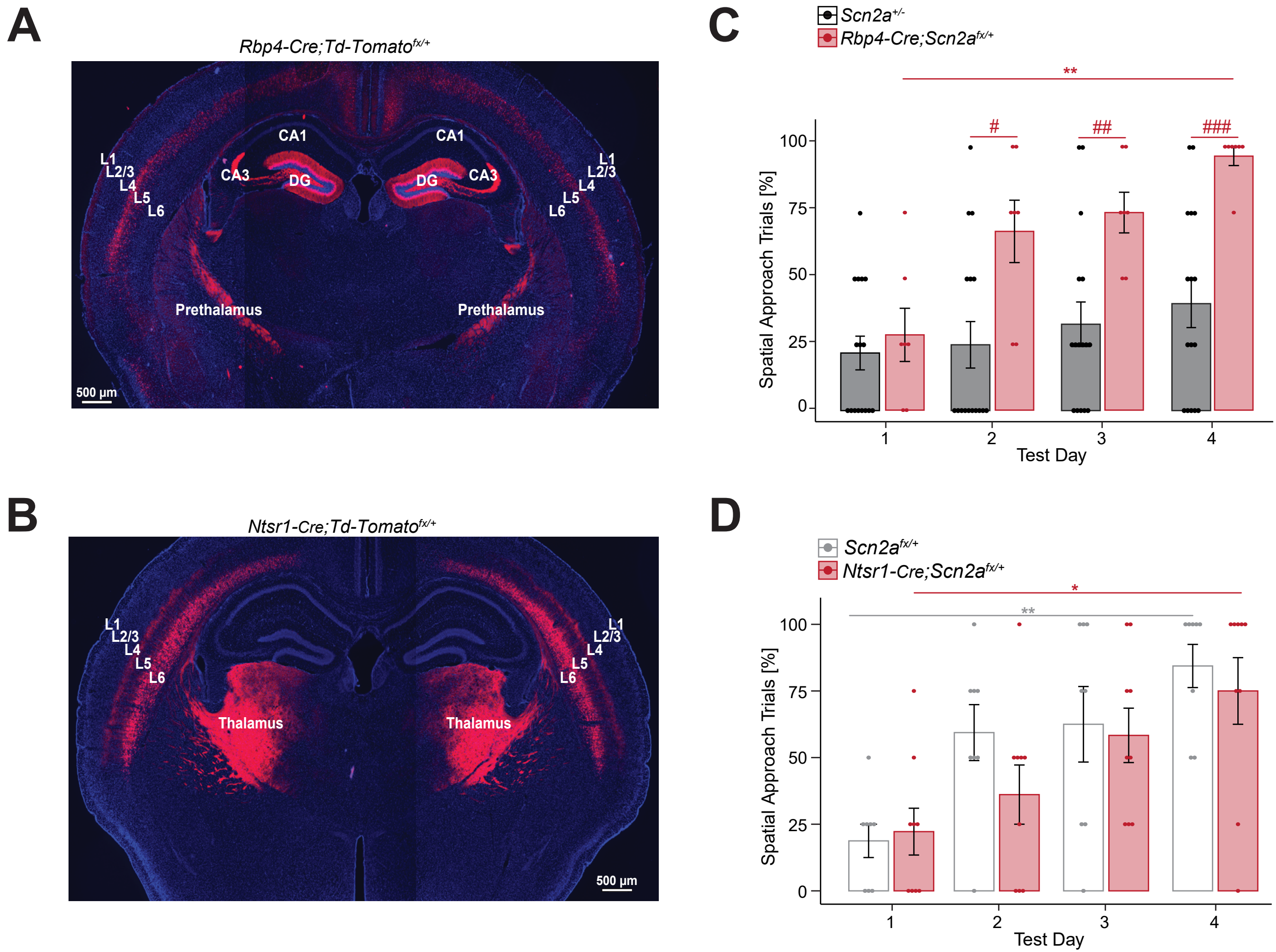

Supplemental Fig. 4: Reduced *Scn2a* expression in cortical layer 5 or 6 does not impair spatial learning.

(A) Image of coronal brain section with *Rbp4-Cre* recombination (red staining) in cortical layer(L) 5, the dentate gyrus and CA3 in *Rbp4-Cre;Td-Tomato*$^{fx/+}$ reporter mice, with DAPI as nuclear counterstain (blue staining).

(B) Image of coronal brain section with *Ntsr1-Cre* recombination (red staining) in cortical L6, in *Ntsr1-Cre;Td-Tomato*$^{fx/+}$ reporter mice, with DAPI as nuclear counterstain (blue staining).

(C) Scatter bar plot of spatial search trials in *Rbp4-Cre;Scn2a*$^{fx/+}$ (n = 7) and *Scn2a*$^{+/-}$ (n = 16) mice across test days in the BM. Points represents individual mice; error bars indicate mean ± SEM. Friedman test with Dunn's post-hoc test and two-way RM ANOVA with a Tukey post-hoc test. Friedman test: $p = 0.2434$ (*Scn2a*$^{+/-}$) and $p = 0.0059$ (*Rbp4-Cre;Scn2a*$^{fx/+}$). **(red) $p = 0.0080$. RM ANOVA: Effect of genotype: $p = 0.0012$; effect of test day: $p < 0.0001$; interaction: $p = 0.0216$. ###p < 0.0001. ##p = 0.0065. #p = 0.0489.

(D) Same as (**C**) but for *Ntsr1-Cre;Scn2a*$^{fx/+}$ (n = 9) and *Scn2a*$^{fx/+}$ (n = 8) mice. Friedman test: $p = 0.0058$ (*Scn2a*$^{fx/+}$) and $p = 0.0075$ (*Ntsr1-Cre;Scn2a*$^{fx/+}$). *(red) $p = 0.0155$. **(gray) $p = 0.0060$. RM ANOVA: No effect of genotype: $p = 0.4503$; effect of test day: $p < 0.0001$.

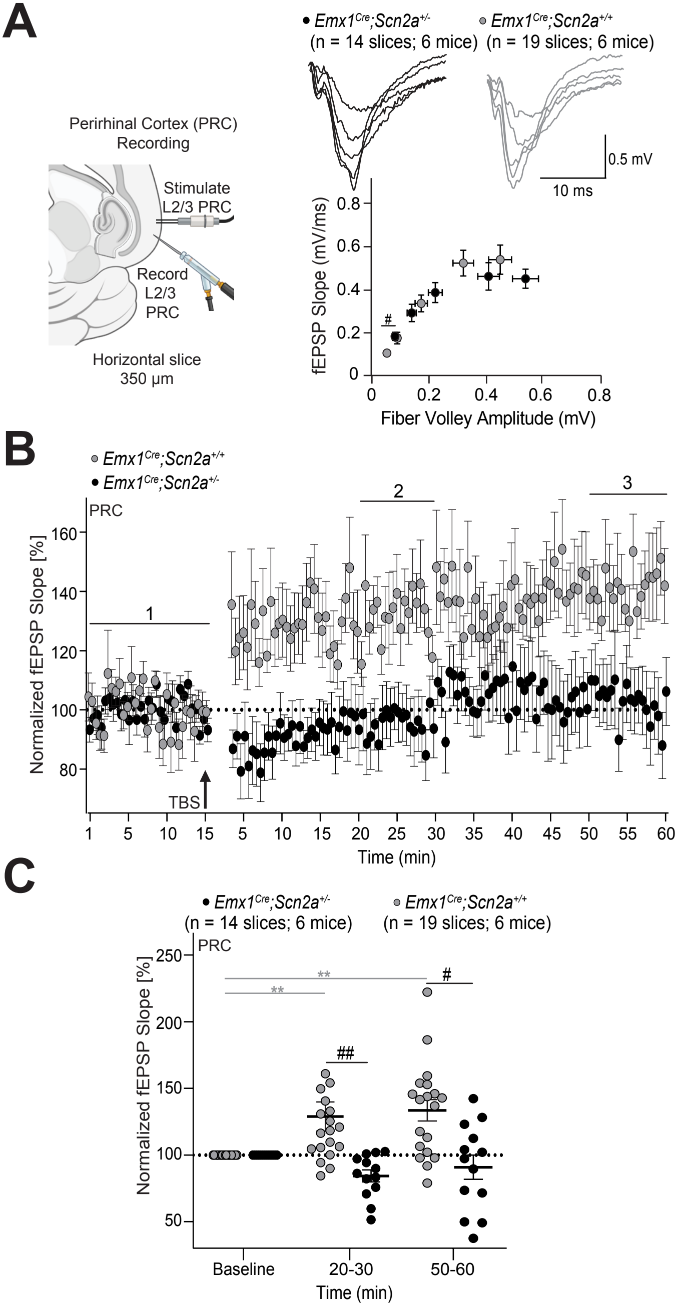

Supplemental Fig. 5: A global reduction of *Scn2a* in the cortex is associated with impaired perirhinal cortex LTP and spatial learning.
(A) Recording paradigm in PRC. I-O for fEPSP slope in *Emx1^{Cre};Scn2a$^{fx/+}$* and *Emx1^{Cre};Scn2a$^{+/+}$* mice. Inset shows representative fEPSPs. No effect of genotype: $p = 0.7300$; effect of stimulus intensity: $p < 0.0001$; interaction: $p = 0.0049$. #(black) $p = 0.0232$.
(B) Plot of average normalized fEPSP slope over time in brain slices from *Emx1Cre;Scn2a$^{fx/+}$* and *Emx1Cre;Scn2a$^{+/+}$* mice.
(C) Mean normalized fEPSP slope plot at baseline, 30- and 60-minutes post-TBS. Each point represents an individual brain slice; bars denote mean ± SEM. Effect of genotype: $p = 0.0047$; effect of time: $p = 0.0284$; interaction: $p = 0.0056$. **(gray) $p = 0.0063$ and $p = 0.0017$, respectively. ##$p = 0.0021$. #$p = 0.0290$.

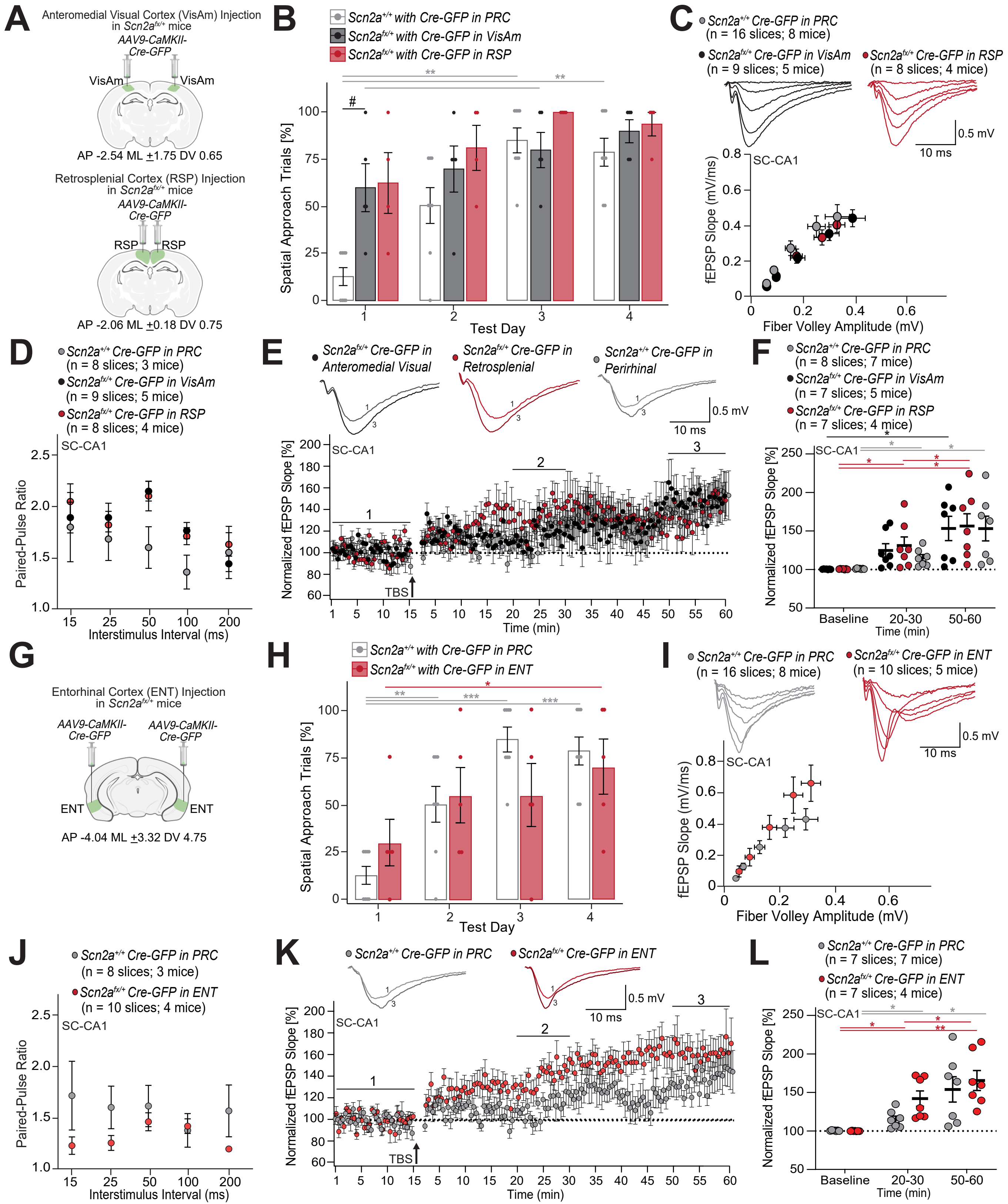

A
Anteromedial Visual Cortex (VisAm) Injection in Scn2a fx/+ mice
AAV9-CaMKII-Cre-GFP
VisAm
VisAm
AP -2.54 ML ±1.75 DV 0.65
Retrosplenial Cortex (RSP) Injection in Scn2a fx/+ mice
AAV9-CaMKII-Cre-GFP
RSP
RSP
AP -2.06 ML ±0.18 DV 0.75
B
Scn2a +/+ with Cre-GFP in PRC
Scn2a fx/+ with Cre-GFP in VisAm
Scn2a fx/+ with Cre-GFP in RSP
Spatial Approach Trials [%]
Test Day
C
Scn2a +/+ Cre-GFP in PRC (n = 16 slices; 8 mice)
Scn2a fx/+ Cre-GFP in VisAm (n = 9 slices; 5 mice)
Scn2a fx/+ Cre-GFP in RSP (n = 8 slices; 4 mice)
0.5 mV
10 ms
SC-CA1
fEPSP Slope (mV/ms)
Fiber Volley Amplitude (mV)
D
Scn2a +/+ Cre-GFP in PRC (n = 8 slices; 3 mice)
Scn2a fx/+ Cre-GFP in VisAm (n = 9 slices; 5 mice)
Scn2a fx/+ Cre-GFP in RSP (n = 8 slices; 4 mice)
SC-CA1
Paired-Pulse Ratio
Interstimulus Interval (ms)
E
Scn2a fx/+ Cre-GFP in Anteromedial Visual
Scn2a fx/+ Cre-GFP in Retrosplenial
Scn2a +/+ Cre-GFP in Perirhinal
0.5 mV
10 ms
SC-CA1
Normalized fEPSP Slope [%]
TBS
Time (min)
F
Scn2a +/+ Cre-GFP in PRC (n = 8 slices; 7 mice)
Scn2a fx/+ Cre-GFP in VisAm (n = 7 slices; 5 mice)
Scn2a fx/+ Cre-GFP in RSP (n = 7 slices; 4 mice)
SC-CA1
Normalized fEPSP Slope [%]
Baseline
20-30
50-60
Time (min)
G
Entorhinal Cortex (ENT) Injection in Scn2a fx/+ mice
AAV9-CaMKII-Cre-GFP
AAV9-CaMKII-Cre-GFP
ENT
ENT
AP -4.04 ML ±3.32 DV 4.75
H
Scn2a +/+ with Cre-GFP in PRC
Scn2a fx/+ with Cre-GFP in ENT
Spatial Approach Trials [%]
Test Day
I
Scn2a +/+ Cre-GFP in PRC (n = 16 slices; 8 mice)
Scn2a fx/+ Cre-GFP in ENT (n = 10 slices; 5 mice)
0.5 mV
10 ms
SC-CA1
fEPSP Slope (mV/ms)
Fiber Volley Amplitude (mV)
J
Scn2a +/+ Cre-GFP in PRC (n = 8 slices; 3 mice)
Scn2a fx/+ Cre-GFP in ENT (n = 10 slices; 4 mice)
SC-CA1
Paired-Pulse Ratio
Interstimulus Interval (ms)
K
Scn2a +/+ Cre-GFP in PRC
Scn2a fx/+ Cre-GFP in ENT
0.5 mV
10 ms
SC-CA1
Normalized fEPSP Slope [%]
TBS
Time (min)
L
Scn2a +/+ Cre-GFP in PRC (n = 7 slices; 7 mice)
Scn2a fx/+ Cre-GFP in ENT (n = 7 slices; 4 mice)
SC-CA1
Normalized fEPSP Slope [%]
Baseline
20-30
50-60
Time (min)

Supplemental Fig. 6: Reduction of Scn2a in the anteromedial visual cortex, retrosplenial cortex, and entorhinal cortex is insufficient to impair spatial learning in the Barnes maze.

(A) Approach for *AAV9-CaMKII-Cre-GFP* (*Cre-GFP*) viral-mediated reduction of *Scn2a* in the anteromedial visual cortex (VisAm) and retrosplenial cortex (RSP) of $Scn2a^{fx/+}$ mice.

(B) Scatter bar plot of spatial trials for $Scn2a^{+/+}$ mice with *Cre-GFP* in PRC (n = 8), $Scn2a^{fx/+}$ mice with *Cre-GFP* in VisAm (n = 5) and in RSP (n = 4) in the BM. Friedman test with Dunn's post-hoc test: p = 0.2106 ($Scn2a^{fx/+}$ *Cre-GFP* in VisAm), p = 0.2812 ($Scn2a^{fx/+}$ *Cre-GFP* in RSP) and p = 0.0002 ($Scn2a^{+/+}$ *Cre-GFP* in PRC). **(gray) p = 0.0020 and p = 0.0042, respectively. Two-way RM ANOVA with Tukey post-hoc test: Effect of condition: p = 0.0102; test day: p < 0.0001. #(black) p = 0.0375.

(C) Inset shows representative fEPSPs. I-O curve in SC-CA1 for $Scn2a^{+/+}$ mice with *Cre-GFP* in PRC, $Scn2a^{fx/+}$ mice with *Cre-GFP* in VisAM and in RSP. Two-way RM ANOVA with Tukey post-hoc test. Effect of stimulus intensity: p < 0.0001.

(D) Paired-pulse ratio from $Scn2a^{+/+}$ with *Cre-GFP* in PRC and $Scn2a^{fx/+}$ with *Cre-GFP* in VisAm and in RSP. Two-way RM ANOVA with Fisher's LSD post-hoc test. Effect of interstimulus interval: p = 0.0006.

(E) Plot of average normalized fEPSP slope in slices from $Scn2a^{+/+}$ with *Cre-GFP* in PRC and $Scn2a^{fx/+}$ with *Cre-GFP* in VisAm and RSP.

(F) Mean normalized fEPSP slope plot at baseline, 30- and 60-minutes post-TBS. Each point represents an individual brain slice; bars denote mean ± SEM. Effect of time: p < 0.0001. *(black) p = 0.0178. *(gray) p = 0.0311 and p = 0.0400, respectively. *(red) p = 0.0184. p = 0.0162, and p = 0.0435, respectively.

(G) Approach for *Cre-GFP* viral-mediated reduction of *Scn2a* in the entorhinal cortex (ENT) of $Scn2a^{fx/+}$ mice.

(H) Scatter bar plot of spatial trials for $Scn2a^{+/+}$ mice with *Cre-GFP* in PRC (n = 8) and $Scn2a^{fx/+}$ mice with *Cre-GFP* in ENT (n = 5) in the BM. Friedman test with Dunn's post-hoc test: p = 0.0851 ($Scn2a^{fx/+}$ mice with *Cre-GFP* in ENT) and p = 0.0002 ($Scn2a^{+/+}$ mice with *Cre-GFP* in PRC). **(gray) p = 0.0020 and p = 0.0042, respectively. *(red) p = 0.0275. Two-way RM ANOVA with Holms-Sidak post-hoc test: Effect of test day: p < 0.0001; interaction between condition and test day: p = 0.0114. This interaction is characterized by $Scn2a^{+/+}$ mice with *Cre-GFP* in PRC increasing in spatial trials across time more quickly than $Scn2a^{fx/+}$ mice with *Cre-GFP* in ENT, likely due to smaller sample size.

(I) Inset shows representative fEPSPs for each condition. I-O curve in SC-CA1 for $Scn2a^{+/+}$ with *Cre-GFP* in PRC and $Scn2a^{fx/+}$ with *Cre-GFP* in ENT. Two-way RM ANOVA with Tukey post-hoc test. Effect of stimulus intensity: p < 0.0001.

(J) Paired-pulse ratio from $Scn2a^{+/+}$ with *Cre-GFP* in PRC and $Scn2a^{fx/+}$ with *Cre-GFP* in ENT. Two-way RM ANOVA with Fisher's LSD post-hoc test.

(K) Plot of average normalized fEPSP slope in slices from $Scn2a^{+/+}$ with *Cre-GFP* in PRC and $Scn2a^{fx/+}$ with *Cre-GFP* in ENT.

(L) Mean normalized fEPSP slope plot at baseline, 30- and 60-minutes post-TBS. Each point represents an individual brain slice; bars denote mean ± SEM. Effect of time: p < 0.0001. *(gray) p = 0.0311 and p = 0.0400, respectively. *(red) p = 0.0132 and p = 0.0490, respectively. **(red) p = 0.0056.

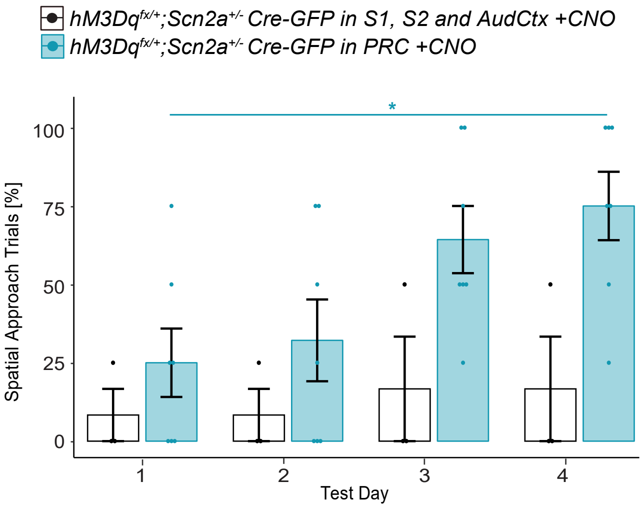

Supplemental Fig. 7: Off-target viral injections in *hM3Dq*$^{fx/+}$*; Scn2a*$^{+/-}$ mice fail to rescue spatial learning behavior.

When *CaMKII-Cre-GFP* virus is injected in regions outside of the PRC (n = 3 in specifically S1, S2, and Auditory Cortex), spatial learning remains at *Scn2a*$^{+/-}$ levels in *hM3Dq*$^{fx/+}$*;Scn2a*$^{+/-}$ injected mice. Statistical analyses were performed at α= 0.05. Friedman test with Dunn's post-hoc test: *p = 0.0056 (on-target) and p > 0.9999 (off-target). *(teal) p = 0.0312. RM ANOVA with Holms-Sidak post-hoc test: effect of targeting: p = 0.0359; effect of test day: p = 0.0496; interaction between test day and targeting: p = 0.2097.

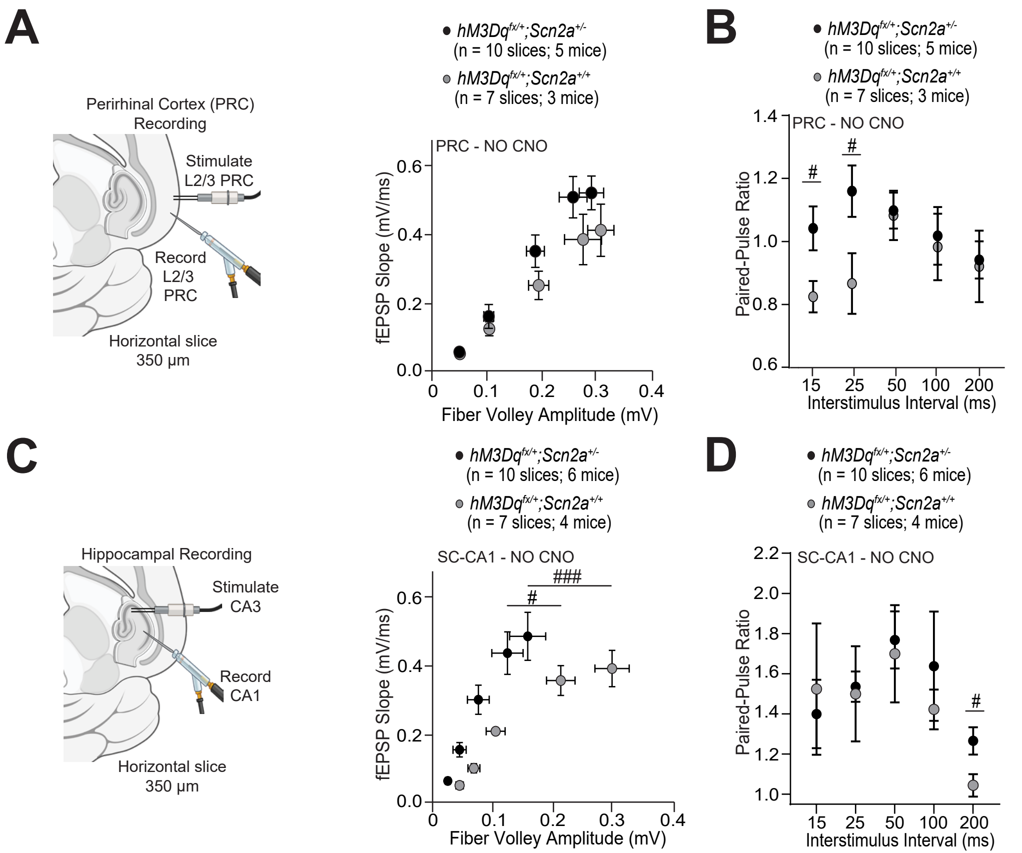

Supplemental Fig. 8: *hM3Dq$^{fx/+}$;Scn2a$^{+/-}$* mice without CNO replicate the reduced PRC release probability and SC-CA1 baseline synaptic transmission observed in *Scn2a$^{+/-}$* mice.

(A) Left: Recording paradigm in PRC. Right: I-O curve for fEPSP slope in slices from *hM3Dq$^{fx/+}$;Scn2a$^{+/-}$* and *hM3Dq$^{fx/+}$;Scn2a$^{+/+}$* mice. Effect of stimulation intensity: $p < 0.0001$.
(B) Paired-pulse ratio between conditioning and test fEPSP slope in slices from *hM3Dq$^{fx/+}$;Scn2a$^{+/-}$* and *hM3Dq$^{fx/+}$;Scn2a$^{+/+}$* mice. Effect of genotype: $p = 0.0214$; #p = 0.0152 and p = 0.0415, respectively.
(C) Left: Recording paradigm in SC-CA1. Right: I-O curve for fEPSP slope in slices from *hM3Dq$^{fx/+}$;Scn2a$^{+/-}$* and *hM3Dq$^{fx/+}$;Scn2a$^{+/+}$* mice. Effect of genotype: $p = 0.0218$; effect of stimulation intensity: $p < 0.0001$; interaction: $p < 0.0001$. ###(FV) $p < 0.0001$. #(FV) $p = 0.0148$.
(D) Paired-pulse ratio between conditioning and test fEPSP slope in slices from *hM3Dq$^{fx/+}$;Scn2a$^{+/-}$* and *hM3Dq$^{fx/+}$;Scn2a$^{+/+}$* mice. Effect of interstimulus interval: $p = 0.0439$. # $p = 0.0238$.
Statistical analysis computed at $\alpha = 0.05$ with two-way RM-ANOVA with Tukey post-hoc test (**A**, **C**) or Fisher's LSD (**B**, **D**).
